# Mutational re-modeling of di-aspartyl intramembrane proteases: uncoupling physiologically-relevant activities from those associated with Alzheimer’s disease

**DOI:** 10.18632/oncotarget.18299

**Published:** 2017-05-30

**Authors:** Anastasia P. Grigorenko, Youri K. Moliaka, Olga V. Plotnikova, Alexander Smirnov, Vera A. Nikishina, Andrey Y. Goltsov, Fedor Gusev, Tatiana V. Andreeva, Omar Nelson, Ilya Bezprozvanny, Evgeny I. Rogaev

**Affiliations:** ^1^ Department of Psychiatry, Brudnick Neuropsychiatric Research Institute, University of Massachusetts Medical School, Worcester, MA 01604, USA; ^2^ Department of Genomics and Human Genetics, Institute of General Genetics, Russian Academy of Sciences, Moscow, 119991 Russia; ^3^ Center for Brain Neurobiology and Neurogenetics, Institute of Cytology and Genetics, Siberian Branch of the Russian Academy of Sciences, Novosibirsk, 630090, Russia; ^4^ Department of Physiology, University of Texas Southwestern Medical Center at Dallas, Dallas, TX 75390-9040, USA; ^5^ Center for Genetics and Genetic Technologies, Faculty of Biology, Faculty of Bioengineering and Bioinformatics, Lomonosov Moscow State University, Moscow, 119234, Russia

**Keywords:** regulated intramembrane proteolysis, intramembrane aspartyl proteases, presenilin, IMPAS/SPP, mutational re-modelling

## Abstract

The intramembrane proteolytic activities of presenilins (PSEN1/PS1 and PSEN2/PS2) underlie production of β-amyloid, the key process in Alzheimer’s disease (AD). Dysregulation of presenilin-mediated signaling is linked to cancers. Inhibition of the γ-cleavage activities of PSENs that produce Aβ, but not the ε-like cleavage activity that release physiologically essential transcription activators, is a potential approach for the development of rational therapies for AD. In order to identify whether different activities of PSEN1 can be dissociated, we designed multiple mutations in the evolutionary conserved sites of PSEN1. We tested them *in vitro* and *in vivo* assays and compared their activities with mutant isoforms of presenilin-related intramembrane di-aspartyl protease (IMPAS1 (IMP1)/signal peptide peptidase (SPP)). PSEN1 auto-cleavage was more resistant to the mutation remodeling than the ε-like proteolysis. PSEN1 with a G382A or a P433A mutation in evolutionary invariant sites retains functionally important APP ε- and Notch S3- cleavage activities, but G382A inhibits APP γ-cleavage and Aβ production and a P433A elevates Aβ. The G382A variant cannot restore the normal cellular ER Ca^2+^ leak in *PSEN1/PSEN2* double knockout cells, but efficiently rescues the loss-of-function (Egl) phenotype of presenilin in *C. elegans*. We found that, unlike in PSEN1 knockout cells, endoplasmic reticulum (ER) Ca^2+^ leak is not changed in the absence of IMP1/SPP. IMP1/SPP with the analogous mutations retained efficiency in cleavage of transmembrane substrates and rescued the lethality of *Ce-imp-2* knockouts. In summary, our data show that mutations near the active catalytic sites of intramembrane di-aspartyl proteases have different consequences on proteolytic and signaling functions.

## INTRODUCTION

**Figure 1:**
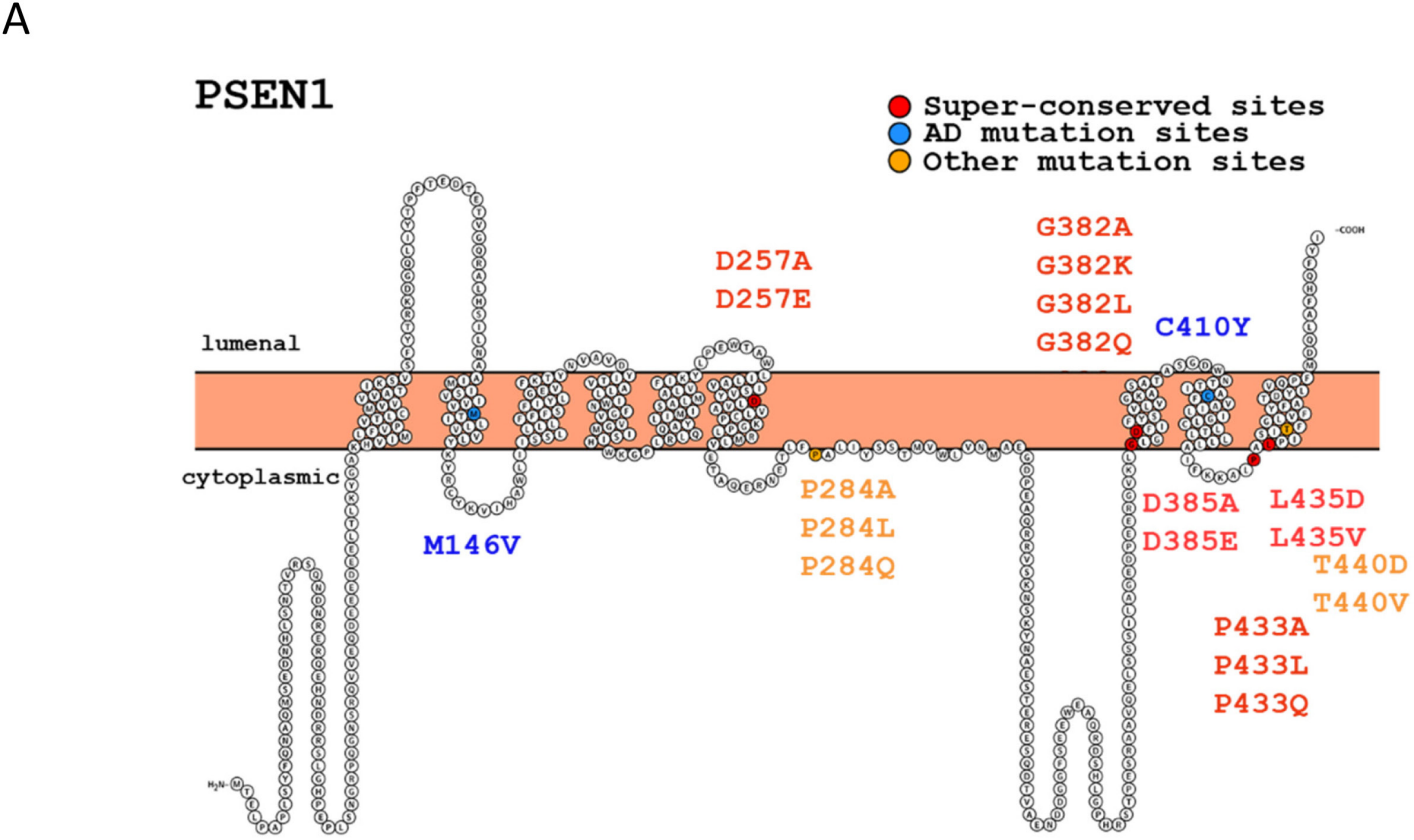

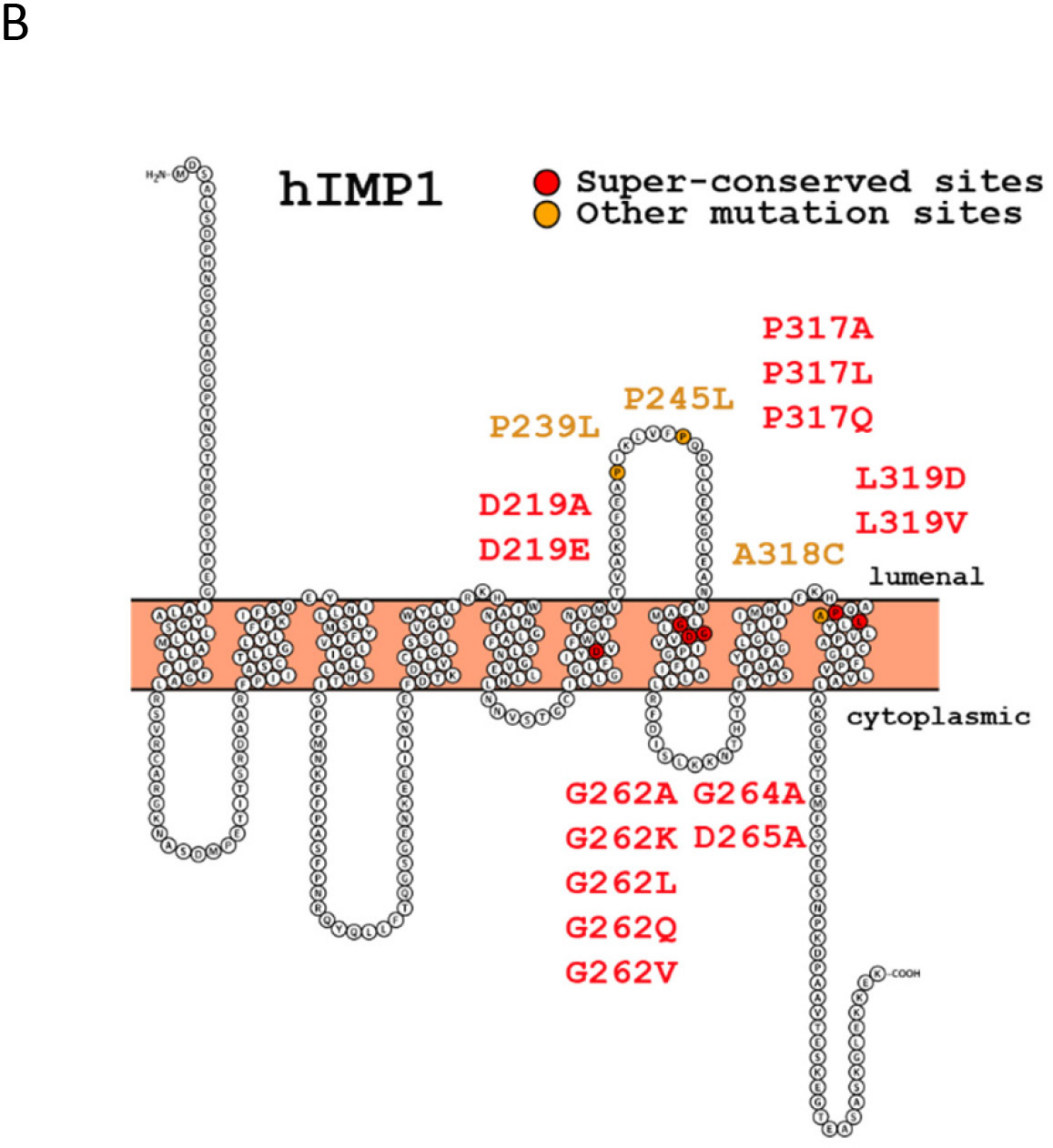
Structures of human presenilin 1 (PSEN1) and IMP1 (hIMP1) proteins and mutations used in the study (Protter program visualization, http://wlab.ethz.ch/protter)

Presenilins (termed PS1 and PS2 or PSEN1 and PSEN2 [1, 2]) are homologous di-aspartyl proteases, capable of cleaving various type I transmembrane proteins within their intramembrane domains. Mutations in *PSEN1* and *PSEN2* are major causative genetic factors of familial cases of Alzheimer’s disease (AD), characterized by early onset AD manifestation [1, 2]. PSEN1 or PSEN2 intramembranous and BACE1 extracellular cleavages of amyloid precursor protein (APP), produce short 40-, 42- amino acid β-amyloid peptides (Aβ). AD autosomal dominant missense mutations in the presenilins have been reported to increase Aβ production and the ratio of Aβ42/40 peptides [3]. PSEN cleavage releases the intracellular domains (ICD) of type I proteins that can act as intracellular signaling molecules, activating gene transcription (e.g., Notch-signaling genes) (reviewed in [3]). Presenilins function as components of the multiple-protein γ-secretase complex and have evolutionarily invariant amino acid signatures around two conserved catalytic aspartates and a PAL-motif (human PSEN1 - D257, D385, PAL433-435) (Figure 1A, Supplementary Figure 1) [4–11]. There are three major proteolytic activities associated with presenilins: (i) “presenilinase”- PSEN autocleavage, (ii) intramembrane γ-cleavage leading to generation of Aβ peptides and (iii) juxtamembrane ε-cleavages of APP, Notch 1 and other type I protein substrates resulting in release of ICDs - intracellular transcriptional regulators (Supplementary Figure 2) Active γ-secretase complex requires four proteins: Nicastrin, PEN2, APH1 and PSEN [12, reviewed in 13, 14]. Although various missense mutations in *PSEN1* lead to autosomal-dominant AD (summarized in AlzForum Mutation Database), heterozygous loss-of-function mutations in *PSEN1* as well as in *Nicastrin* and *PEN2* (haploinsufficiency) have been shown to cause specific severe inflammatory skin disease, termed acne inversa in humans [15], reviewed in [16]. Clinical trials of drugs for AD inhibiting γ-secretase activity revealed various effects on skin, including a higher risk of skin cancer [16, 17]. In mice, loss of *PSEN1* causes skin cancer, and a reduction of PSENs function is responsible for myeloproliferative disease [18, 19]. An inverse association between AD and cancer has been proposed with multiple regulatory mechanisms, including Pin1-, p53-, Wnt-related signaling, proposed to underlie the diseases [20, 21, reviewed in 22]. Among the important presenilin functions is regulation of Wnt signaling/β-catenin phosphorylation and turnover, which can contribute to skin cancer [18, 23–25]. This regulation can occur indirectly via cadherins as described in [26]. Another reported property of PSEN1 is its activity as a low conductance endoplasmic reticulum (ER) Ca^2+^ leak channel with a regulatory role in pathways linked to intracellular Ca^2+^ homeostasis [27–30]. Numerous studies have shown the involvement of PSEN1 in the autophagy-lysosome degradative pathway, which is also a function independent of γ-secretase proteolysis [31–34]. Since both the up- and down-regulation of presenilins and presenilin-mediated signaling pathways, in particular Notch, may lead to various cancers [18, 35–41], the balanced physiological level of presenilin/γ-secretase activity is essential for normal biological function. Therefore, the direct approach for down-regulation of γ-secretase by γ-secretase inhibitors for reduction of Aβ generation may not be appropriate for AD treatment. On the other hand, suppression or modification of proteolytic activity producing Aβ with retained physiological activity of presenilin is an attractive strategy in AD therapy.

The gene family for polytopic proteins termed intramembrane di-aspartyl proteases (IMPAS) or signal peptide proteases (SPP) includes the five known paralogous genes, designated as *IMP1, -2, -3, -4, -5*; or *SPP, SPPL2a, -2b, -2c ,-3*; or *PSH* gene family [42–44]. These proteins were described as structural homologs of presenilins, registered in MEROPS database as peptidase subfamily A22B [45]. PSEN and IMP1/SPP proteases share identical evolutionarily conserved motifs for the catalytic sites YD and GxGD and the PAL domain in their C-termini (Figure 1) [4-7, 42]. Unlike PSEN1 and PSEN2, which cleave type I transmembrane proteins, IMP1/SPP proteins cleave type II transmembrane substrates with the N-terminus oriented to the cytosol [43, 46, 47]. Some potent γ-secretase inhibitors can efficiently work for IMP1/SPP [46]. In our previous work, we showed that human hIMP1/SPP can cleave polytopic PSEN1 protein within its membrane domain *in vitro* [47]*.* In another independent study, a signal peptide peptidase (SPP) that regulates the cleavage of specific short signal peptides in the ER was isolated [43]. The major hIMP1 isoform is identical to human SPP encoded by the same gene and orthologous to *C. elegans Ce-imp-2* gene [42, 43, 47]. We identified a phenotype caused by inactivation of the *Caenorhabditis elegans* hIMP1/SPP orthologue, *Ce-imp-2*, possibly related to a cholesterol-dependent cellular pathway [48]. *In vitro* assays demonstrated that hIMP1/SPP cleaves short signal peptide remnants tethered in ER membranes. This activity may generate short signal sequences that are essential for HLA-E epitopes [43, 49]. This protease also participates in processing of the hepatitis C core protein (HCV core protein, [50], ER-resident tail-anchored proteins, the unfolded protein response (UPR regulator XBP1u and other proteins (data is summarized in Supplementary Figure 3).

Modulating the different activities of PSEN and IMP1/SPP-related di-aspartyl intramembrane proteases is important for a better understanding of their functions and is essential for studies aimed at dissociating their proteolytic properties. Here we created a series of constructs bearing mutations focused upon (i) the highly conserved signature GLGD; (ii) the PAL- motif that is evolutionarily invariant in both the PSEN and IMP1/SPP protein families; and (iii) AD mutation positions of PSEN1 (Figure 1). Various mutations in the same amino acid positions were incorporated to generate subtle or radical alterations of hydrophobicity of the sites [51–53] (Supplementary Tables 1 and 2). We tested how the designed mutations affect the processing and functional properties of PSEN1 enzyme in intramembrane proteolysis assays comparing to similar mutations in the hIMP1/SPP protease (preliminary data were reported in [54]). In addition, for selected mutations, we performed rescue experiments in a transgenic *C. elegans.* To test the effects of the mutations on non-proteolytic functions of PSEN1 and whether IMP1/SPP possess such functions, we employed ER Ca^2+^ leak assays using mouse embryonic fibroblasts deficient in the *PSEN* or *IMP1/SPP* genes.

## RESULTS

### Proteolytic properties of PSEN1

#### (1) Presenilinase auto-cleavage

Presenilin proteins undergo several post-translation modifications including endoproteolysis of full length protein (FL PSEN) by “presenilinase” within the seventh hydrophobic domain leading to accumulation of 27-28 kDa N-terminal (NTF) and 16-17 kDa C-terminal (CTF) products [55]. “Presenilinase” is likely to be the presenilin *per se* undergoing autoprocessing in the cis-position that has aspartyl protease activity distinct from γ-secretase biochemical properties. The proteolytic event takes place mainly in ER compartments and requires at last one other γ-secretase complex component, PEN2 protein [56–60]. Mutations in conserved aspartates of PSEN1 are critical for both presenilinase and γ-secretase activities. Over-expression of NTF or CTF fragments bearing mutations at catalytic aspartate sites cannot restore the γ-secretase activity in *C. elegans* mutants [61]. Mutations in the “presenilinase” cleavage site (PSEN1 M292D, M292E) strongly inhibit the autocleavage while the AD-related PSEN1ΔE9 mutation completely abolishes PSEN1 endoproteolysis, although in both cases γ-secretase remains active [59, 62]. Variable inhibitory effects on PSEN1 processing have been described for different AD-associated mutations [63–65].

**Figure 2:**
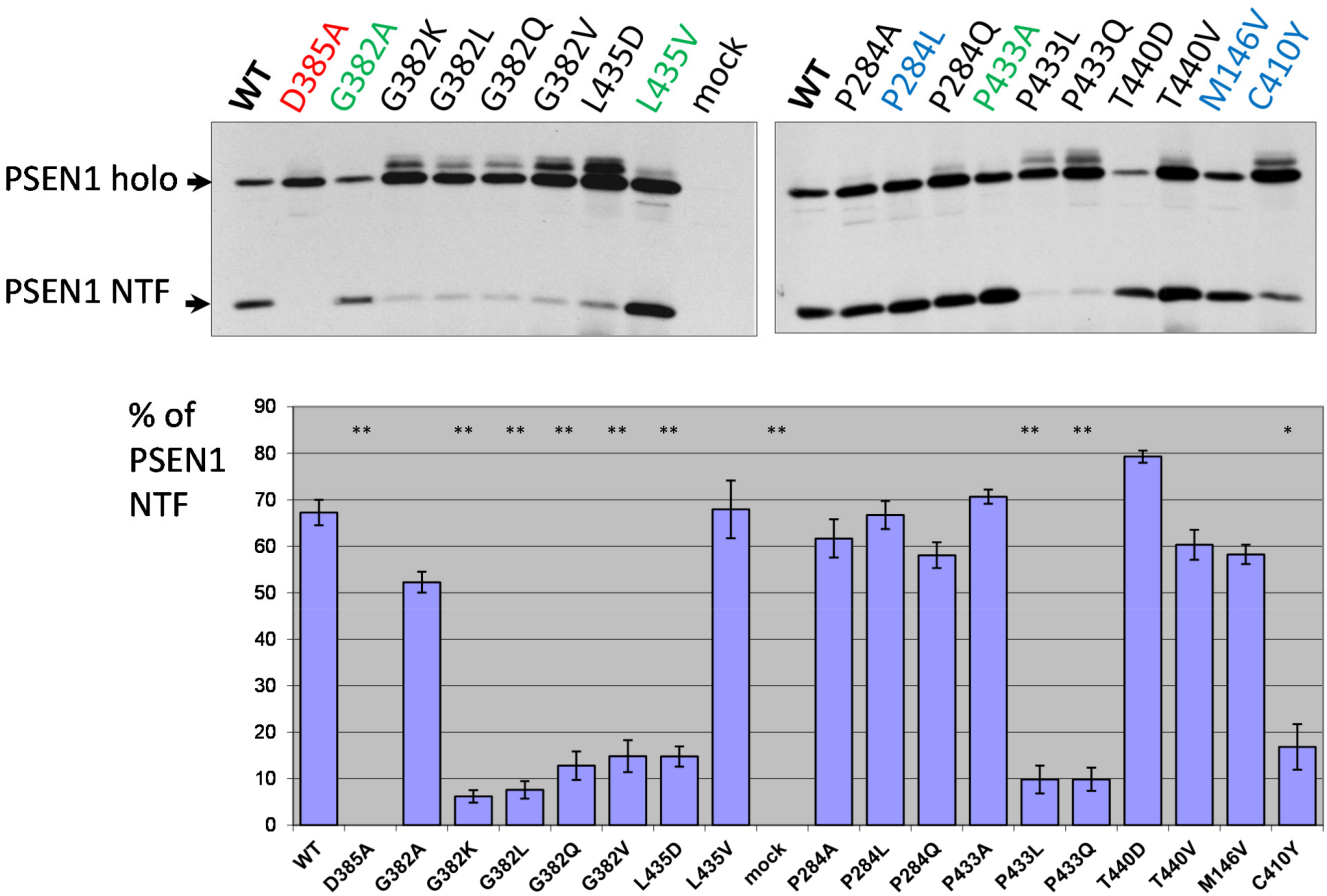
Study of PSEN1 mutants in presenilinase cleavage assay The cleavage product was detected by PSEN1 N-terminal antibody. Transfections were performed in *PSEN1*^*-/-*^*/PSEN2*^*-/-*^ MEF cells. Histogram shows the percent ratio of PSEN1 NTF to uncleaved PSEN1 holoprotein. Error bars represent SE. In this and all other Figures, mutations in super-conserved aspartates are indicated in red, mutations with similar amino acid changes, which do not alter the physicochemical properties of the site, are in green and AD mutations are in blue. P-value significance codes: ‘**’ <0.01, ‘*’ <0.05.

We transfected *PSEN1*^*-/-*^*/PSEN2*^*-/-*^ mouse embryonic fibroblasts (MEF) cells with wild type or mutant PSEN1 constructs and examined PSEN1 auto-cleavage products via immunoblotting using PSEN1 N-terminal antibody (Figure 2). We confirmed previous findings [4, 6] that mutations in specific aspartate residues of PSEN1 (D257A, D257E, D385A, D385E) completely abolish PSEN holoprotein cleavage as well as γ-secretase activity. We used cells over-expressing PSEN1 D385A as a dominant-negative control in all proteolytic assays. Our data on the effects of *PSEN1* mutations indicate that mutations that critically change physicochemical properties (hydrophobicity) of the ultra-conserved amino acids G382, P433 and L435, strongly inhibit, but not completely abolish, the presenilinase activity. Similar amino acid substitutions G382A, P433A and L435V in the evolutionary conserved sites had much less or no effect on PSEN1 processing (Figure 2, Supplementary Table 1). We observed similar weak inhibitory effects of the various mutations in less conserved and AD-related amino acid positions (Figure 2). As previously described, AD-associated C410Y mutation led to a strong inhibition of PSEN1 processing (Figure 2) [64, 65].

#### (2) Notch1ΔE cleavage by PSEN1

Among the major PSEN substrates is a Notch family of proteins, which mediate important signaling pathways in the development and function of multicellular organisms [66]. PSEN1 has been shown to cleave NH^2^-terminally-truncated Notch derivates (NotchΔE) within their intramembrane domains (ε- or S3-cleavage) and to release the Notch intracellular domain (NICD), which functions as a transcriptional activator[67–70].

**Figure 3:**
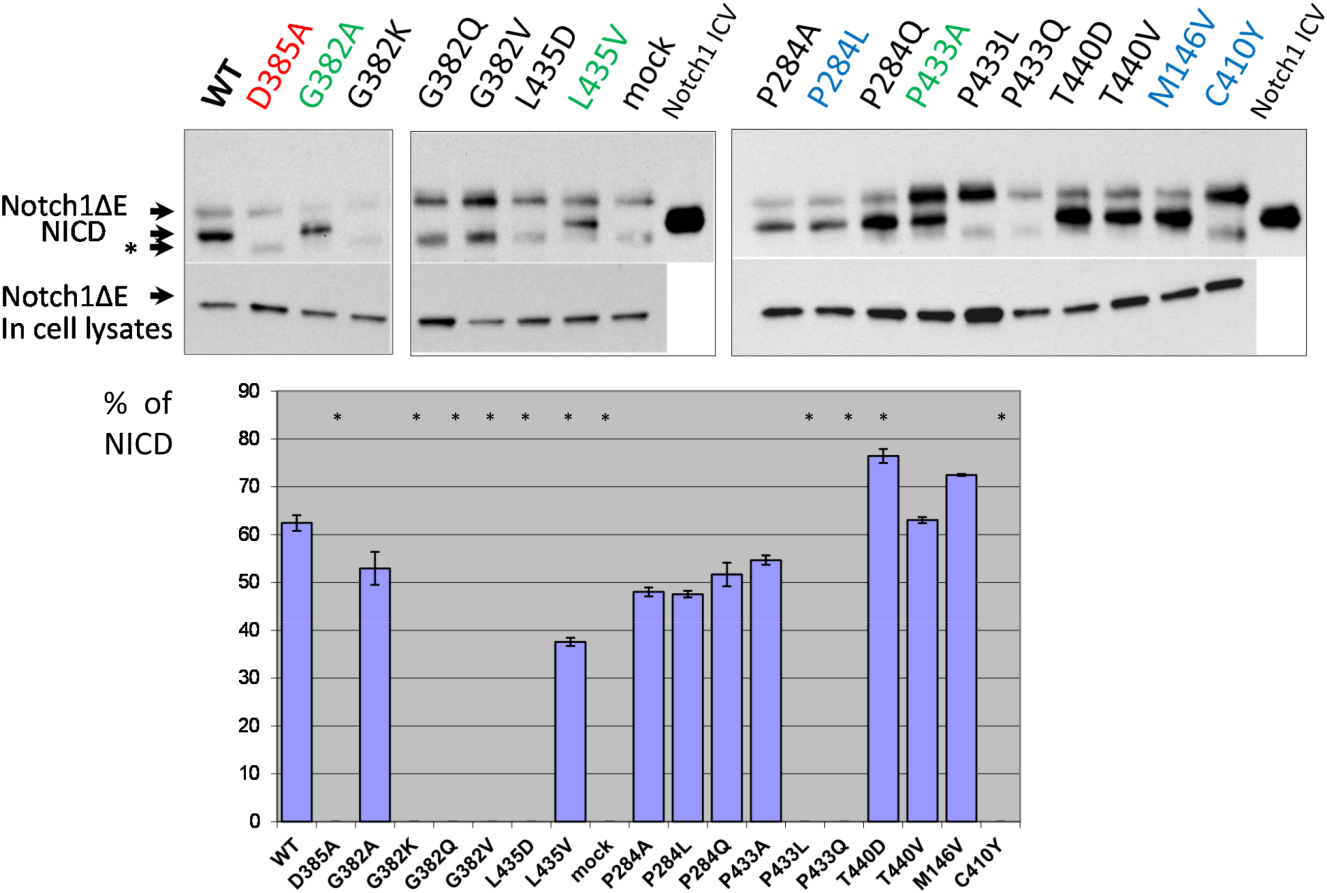
Study of PSEN1 mutants in Notch1 proteolytic assay Co-transfections of wild type or mutant PSEN1 and NotchΔE constructs were performed in *PSEN1*^*-/-*^*/PSEN2*^*-/-*^ MEF cells. Cleavage products were detected by C-myc antibody. Histogram shows the percent ratio of NICD to uncleaved NotchΔE. An additional nonspecific band is marked by an asterisk. P-value significance codes: ‘*’ <0.05.

We co-transfected Notch1ΔE along with different *PSEN1* mutant constructs in *PSEN1*^*-/-*^*/PSEN2*^*-/-*^ MEF cells and studied the cleavage products in cell-free membrane assays (see Material and Methods). Consistent with the data from the “presenilinase” cleavage assays, mutations in highly conserved amino acids G382, P433 and L435, which affect the physicochemical properties of the protein, completely suppress Notch1ΔE ε-cleavage, whereas mutations G382A, P433A and L435V had a less inhibitory effect (Figure 3). Among other mutations, including AD-like mutations, only C410Y substitution abolished or strongly inhibited Notch1 processing (Figure 3).

#### (3) Production of carboxy-terminal fragments and Aβ peptides by APP processing

**Figure 4:**
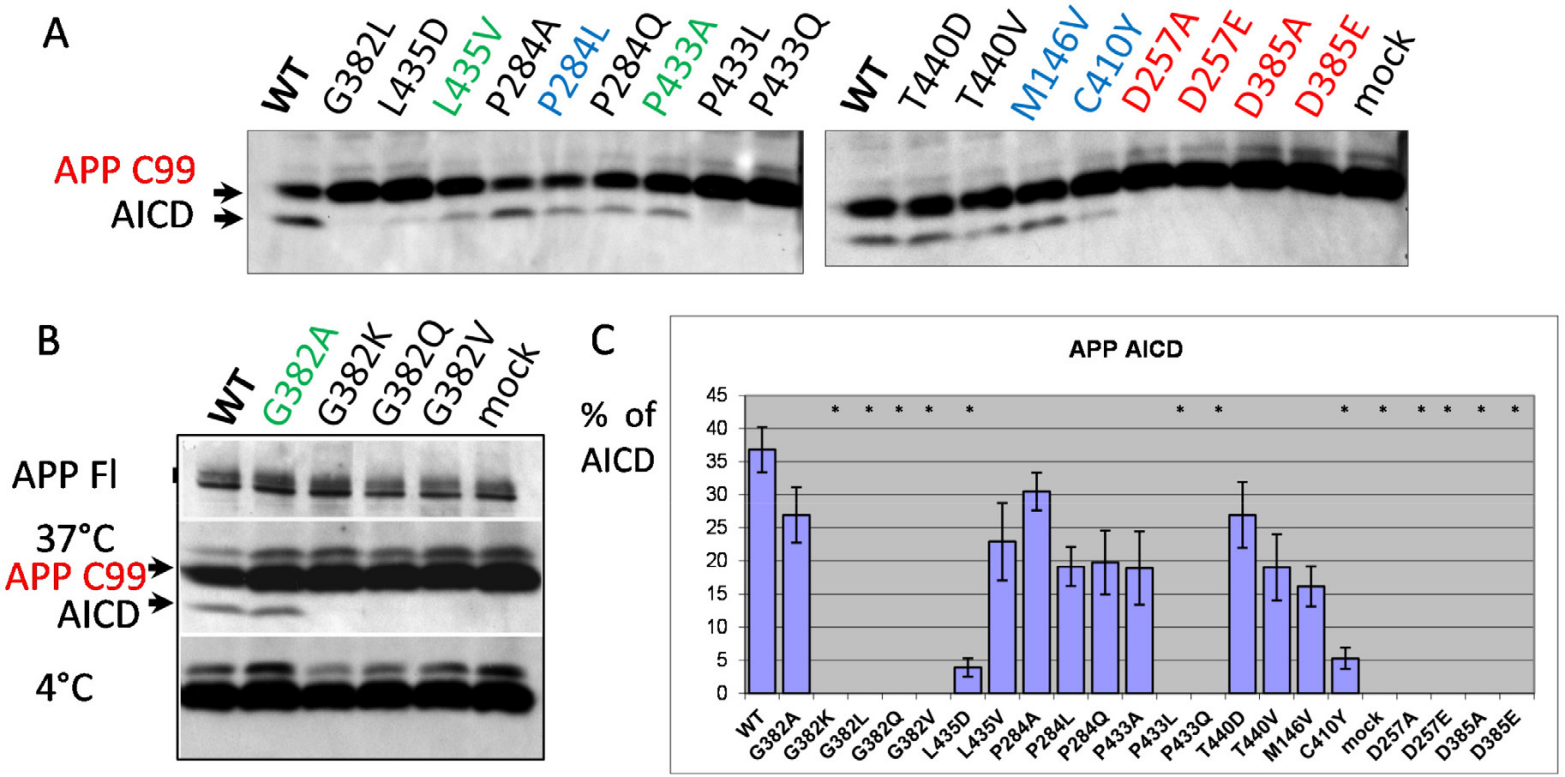
Study of PSEN1 mutants in APP ε-cleavage assay **(A, B)** Co-transfections of wild type or mutant PSEN1 and APP695 bearing the Swedish mutation (ΔNL) were performed in *PSEN1*^*-/-*^*/PSEN2*^*-/-*^ MEF cells. Cell-free intramembrane cleavage was induced by incubation of the samples at 37°C for 2 hours. APP C99 and γ-secretase cleavage product AICD were detected by APP CTF antibody (Sigma). No cleavage was observed for the control samples incubated on ice **(B)**. mAb 22C11 antibody, which detects the full-length APP (APP FL), was used as a transfection control in the assay **(B)**. **(C)** Histogram shows the percent ratio of AICD to uncleaved APP C99. P-value significance codes: ‘*’ <0.05.

Another important type I protein proteolytically processed by presenilins is APP. Extramembrane endoproteolysis of APP by BACE produces the 99-amino acid C-terminus fragment anchored in the membrane. This C-terminus fragment is the substrate for γ-secretase cleavage. We used the transgenic construct APP695 isoform (APP695ΔNL) that bears the Swedish mutation for AD (KM670/671NL), and enhances the overall production of Aβ [71–73] (Supplementary Figure 2C) for co-expression with the mutant *PSEN1* constructs in *PSEN1*^*-/-*^*/PSEN2*^*-/-*^MEF cells. Mutations in PSEN genes are responsible for changes in intramembrane γ-proteolysis events, resulting in accumulation of the amyloidogenic product (Aβ40, Aβ42) that is the major component of amyloid senile plaques in AD [74–76, reviewed in 3]. After shedding of the extracellular N-terminal part of APP, cleavage in the APP ɛ-site by γ-secretase complex generates the carboxy-terminal amyloid intracellular domain (AICD) [77]. AICD, similar to NICD, can regulate transcription of various genes, including the genes encoding EGFR, a protein tyrosine kinase up-regulated in tumors. Reduction of PSEN1 activity can regulate EGFR-mediated tumorogenesis [78]. PSEN1 mutants P284A/L/Q, G382A, L435V, P433A, T440V/D, transfected into *PSEN1*^*-/-*^*/PSEN2*^*-/-*^ MEF cells along with APP695ΔNL, retain their proteolytic capacity to produce AICD showing various inhibitory or no inhibitory effects (Figure 4). No APP ɛ-proteolysis products were detected when D257A/E, D385A/E, G382K/L/Q/V, P433L/Q PSEN1 mutants were transfected into the *PSEN1/PSEN2* double knockout cells. A strong inhibitory effect on APP ɛ-cleavage was demonstrated for mutation in evolutionary conserved site, L435D, and for AD mutation C410Y (Figure 4).

**Figure 5:**
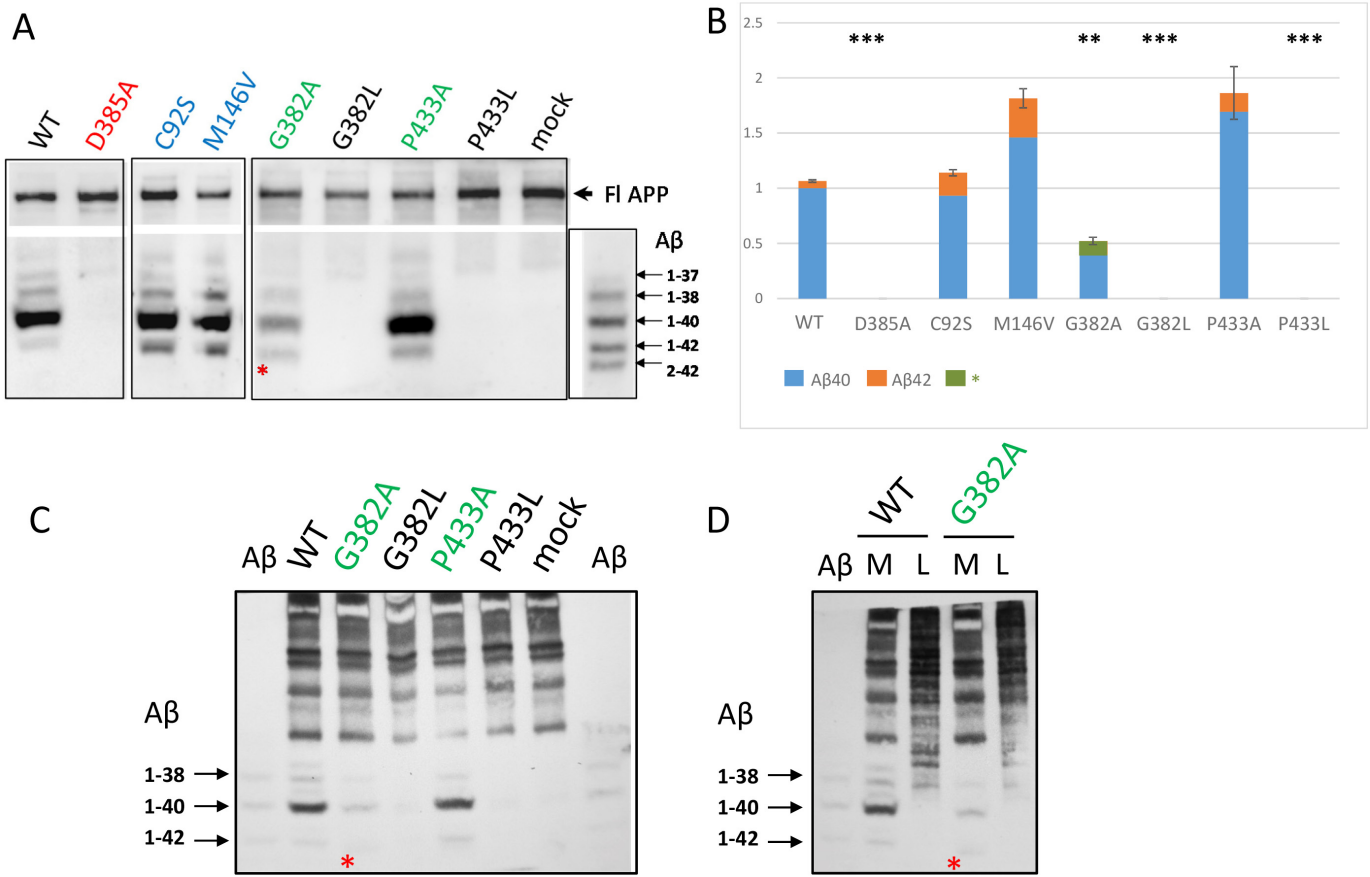
Detection of secreted Aβ peptides in cell culture medium in *PSEN1*^*-/-*^*/PSEN2*^*-/-*^ MEF cells co-transfected with wild type or mutant *PSEN1* and *APP695ΔNL* Immunoprecipitation of secreted Aβ in cell conditioned medium **(A, C, D)** and cell lysates **(D)**. Immunoprecipitated Aβ peptides were detected by 1E8 antibody. Aβ - β-amyloid ladder. M - conditioned medium, L - cell lysate. * - a specific Aβ peptide product, which differs from Aβ42, was detected for the G382A construct. **(B)** Histogram shows the relative ratio of total amyloid (Aβ40 and Aβ42 or * Aβ peptide product) in PSEN1 variants calibrated to wild type Aβ40 level in 2-4 experiments. P-value significance codes: ‘***’ <0.001, ‘**’ <0.01. AD mutations C92S and M146V were used as positive controls for Aβ42 product.

For the mutations in PSEN1 highly conservative sites, we also analyzed the Aβ-peptide profiles. A spectrum of Aβ peptides were immunoprecipitated from *PSEN1*^*-/-*^*/PSEN2*^*-/-*^ MEF cells co-transfected with wild type or mutant PSEN1 and APP695ΔNL constructs. We showed that PSEN1 P433A, that retains Notch1 S3- and APP ɛ-cleavage activities, is also active in APP γ-site cleavage generating elevated level of Aβ peptides. In contrast, for the PSEN1 G382A that shows Notch1 S3- and APP ɛ-cleavage efficacy, we observed a strong inhibition of Aβ40 and other Aβ derivates (Figure 5). PSEN1 D385A, G382L, P433L mutations lead to complete inhibition of Aβ-peptide production compared to wild type PSEN1. The weak intensity low band suggesting the specific Aβ fragment that differs from 1-42 was also observed in assays for PSEN1 G382A using high-resolution SDS-UREA-PAGE electrophoresis. This band was not observed using any other mutant constructs (Figure 5, Supplementary Figure 4).

### Proteolytic properties of IMP1/SPP

#### (1) Presenilin substrate cleavage

Our previous experiments, in which we co-expressed PSEN1 holoprotein and human hIMP1/SPP in cultured cells, revealed that hIMP1/SPP is capable of cleaving a multipass transmembrane PSEN1 protein substrate [47]. We provided evidence that hIMP1/SPP may induce intramembrane proteolysis of PSEN1 in its last hydrophobic domain [47]. The significance of such cleavage activity i*n vivo* has yet to be elucidated but a straightforward immunodetection assay has been developed to monitor proteolytic activity of IMP1 isoforms [47].

**Figure 6:**
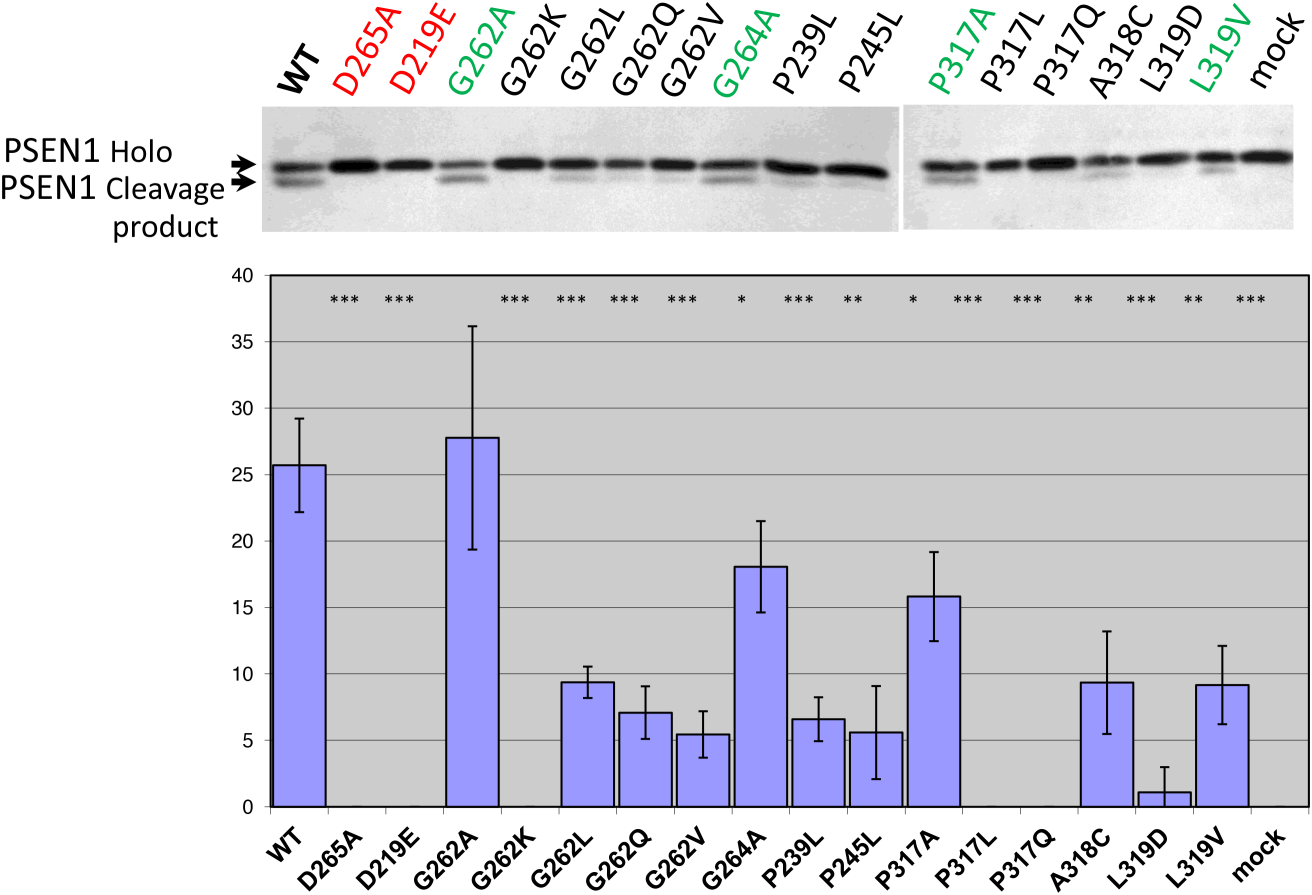
Study of hIMP1 mutants proteolytic cleavage of presenilin substrate Co-transfections of wild type or mutant hIMP1 and PSEN1 constructs were performed in HEK293 cells. Histogram shows the percent ratio of cleaved PSEN1 to uncleaved PSEN1 holoprotein, detected with N-terminal hPSEN1. P-value significance codes: ‘***’ <0.001, ‘**’ <0.01, ‘*’ <0.05.

**Figure 7:**
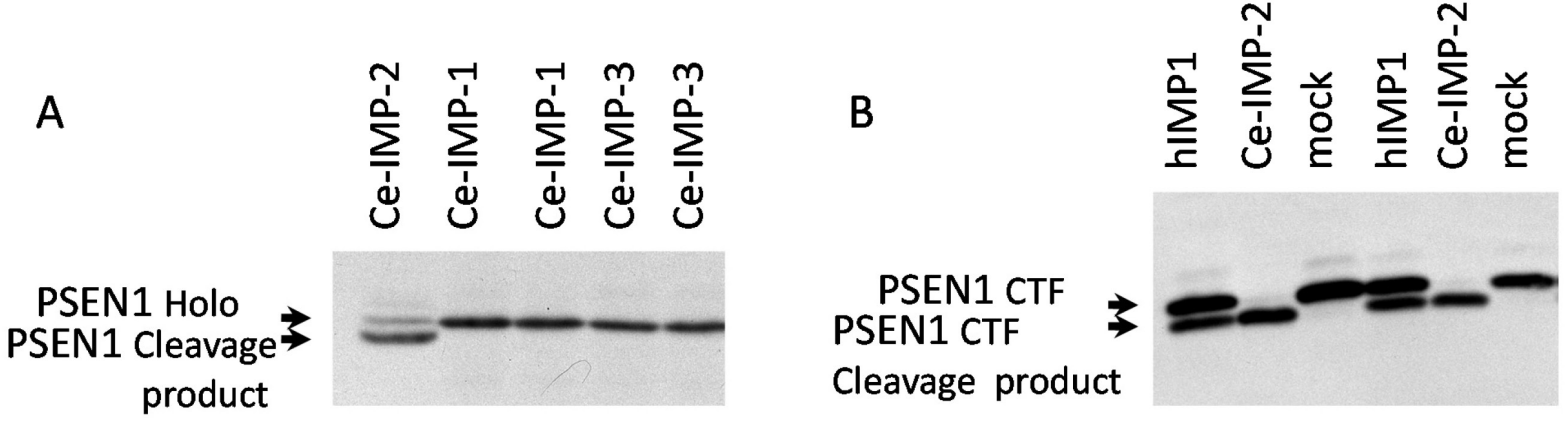
Ce-IMP-2 protein is efficiently cleaving hPSEN1 substrates **(A)** Co-transfections of wild type *C. elegans* IMPAS and human PSEN1 constructs performed in HEK293 cells. Ce-IMP-2 protein, which is an orthologue of human IMP1, but not Ce-IMP-1 or Ce-IMP-3, is capable of cleaving hPSEN1 holoprotein, detected with N-terminal hPSEN1 antibody. **(B)** Co-expression of hPSEN1 CTF and hIMP1 or Ce-IMP-2 protein in *PSEN1*^*-/-*^*, PSEN2*^*-/-*^ double knockout fibroblasts. Efficient cleavage of hPSEN1 C-terminal fragment by Ce-IMP-2, with higher efficiency than by human IMP1 was detected by antibody for an N-terminal epitope of hPSEN1 CTF.

In the present study we co-transfected different IMP1/SPP mutant forms with PSEN1 substrate in HEK293 cells. Substitution mutations to structurally similar amino acids (such as G262A, G264A, P317A and L319V) lead to more efficient rescue capacities than mutations to structurally more distant amino acids in the same positions of hIMP1/SPP (Figure 6, Supplementary Table 2). Any changes in catalytic aspartate residues and distant mutations in evolutionarily conserved sites (e.g. G262K, P317L/Q, L319D) completely or nearly completely inhibited the proteolysis of PSEN1 substrate. The G262L mutation reduced the cleavage of PSEN1 by approximately 40%. We have also shown that Ce-IMP-2, which is the *C. elegans* orthologue of human IMP1/SPP, can also cleave the C-terminal domain of human PSEN1 with even higher efficacy than hIMP1/SPP (Figure 7 and below for more details).

#### (2) HCV substrate cleavage

**Figure 8:**
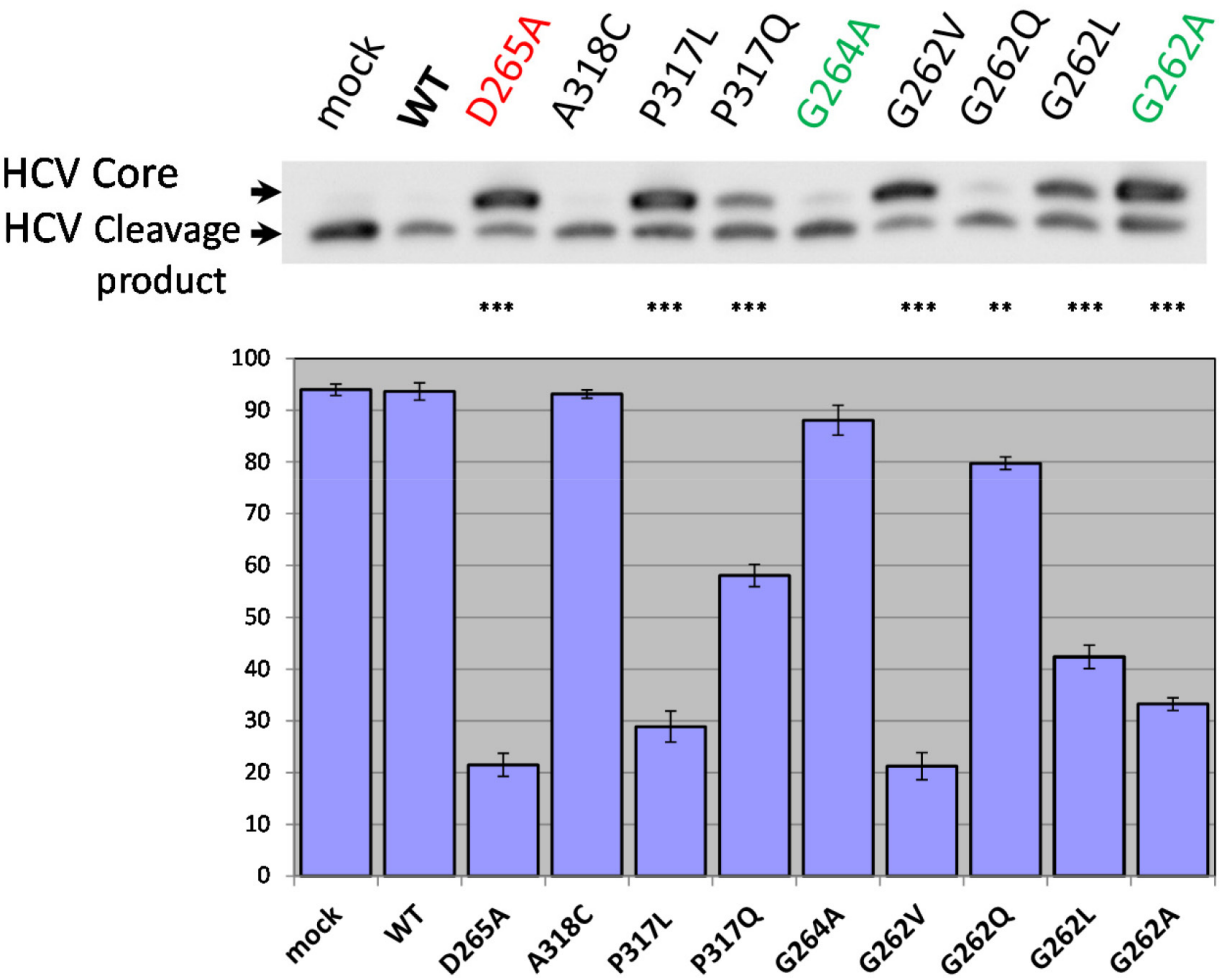
HCV cleavage assay Co-transfections of wild type or mutant hIMP1 and HCV constructs were performed in HEK293 cells. Histogram shows the percent ratio of cleaved HCV to uncleaved HCV core protein, detected by anti-FLAG antibody. P-value significance codes: ‘***’ <0.001, ‘**’ <0.01.

We next examined how wild type and mutant hIMP1/SPP cleave an HCV (hepatitis C virus) substrate (Figure 8). hIMP1/SPP is known to cleave the immature form of HCV core protein in the hydrophobic transmembrane/C-terminal domain and this feature has become incorporated into a commonly used assay to study proteolytic properties of IMP1/SPP [50, 79]. The resulting cleavage product, p21 protein, along with the envelope proteins E1 and E2, are important structural components of the virus capsid [50, 80, 81]. Here we co-transfected FLAG-tagged HCV core protein along with different hIMP1/SPPs and detected the cleavage products in HEK293 cells (Figure 8). There is a strong inhibition of HCV core protein cleavage by hIMP1/SPP mutations D265A, P317L, G262V, and to a lesser extent, by G262L and G262A (Figure 8). Interestingly, hIMP1 G264A and hIMP1 A318C demonstrated high proteolytic activity in the HCV cleavage assay.

### Low conductance ER Ca^2+^leak assay

In addition to the protease function, presenilins may function as low conductance ion channels [27, 28]. Planar lipid bilayer reconstruction techniques showed that wild type presenilin 1 can form a Ca^2+^-permeable ion channel in the ER. Interestingly, mutations in the PSEN1 catalytic aspartate D257A, which is essential for PSEN1 proteolytic function, does not disrupt ion currents; whereas the AD mutation PSEN1 M146V impaired the channel function in a dominant negative manner [27]. The critical pore structural positions were mapped to the PSEN1 hydrophobic domains 7 and 9 [28].

**Figure 9:**
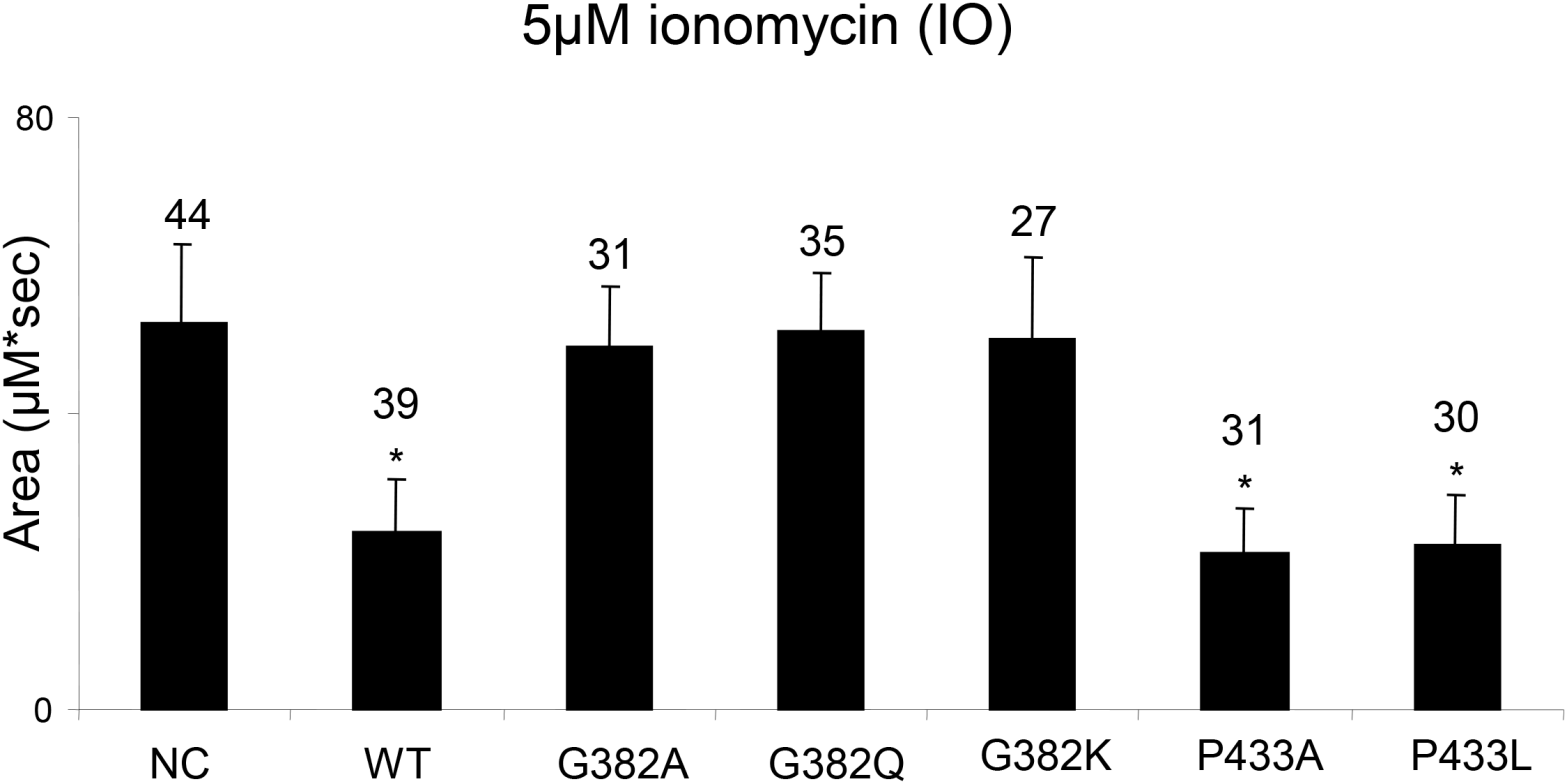
Study of PSEN1 mutants in low conductance ER Ca^2+^ leak assay The histogram shows the mean size of ionomycin (IO)-induced Ca^2+^ pool (±SD) in *PSEN1*^*-/-*^*/PSEN2*^*-/-*^ MEF cells stably transfected with wild type (WT) or mutant PSEN constructs. pEGFP-C3 plasmid was used as a negative control (NC). ‘*’ p<0.05 by ANOVA – significant Ca^2+^ pool changes comparing to NC. The number of cells analyzed is indicated (n).

We next tested whether the mutations in glycine at position 382 (G382) that show variable effects on the proteolytic activities of PSEN1 and located in transmembrane domain 7 affect the cellular ER Ca^2+^ leak in *PSEN1* and *PSEN2* double-knockout MEFs. In a series of experiments, we observed that all tested mutations in glycine in position 382, including the G382A, which retains the γ-secretase activity, were unable to reconstitute the normal ER Ca^2+^leak. In contrast, PSEN1 mutants with substitutions in proline 433 (P433A, P433L) restore the Ca^2+^ leak function in *PSEN1*^-/-^*/PSEN2*^-/-^ double-knockout cells similar to wild type PSEN1 function (Figure 9).

**Figure 10:**
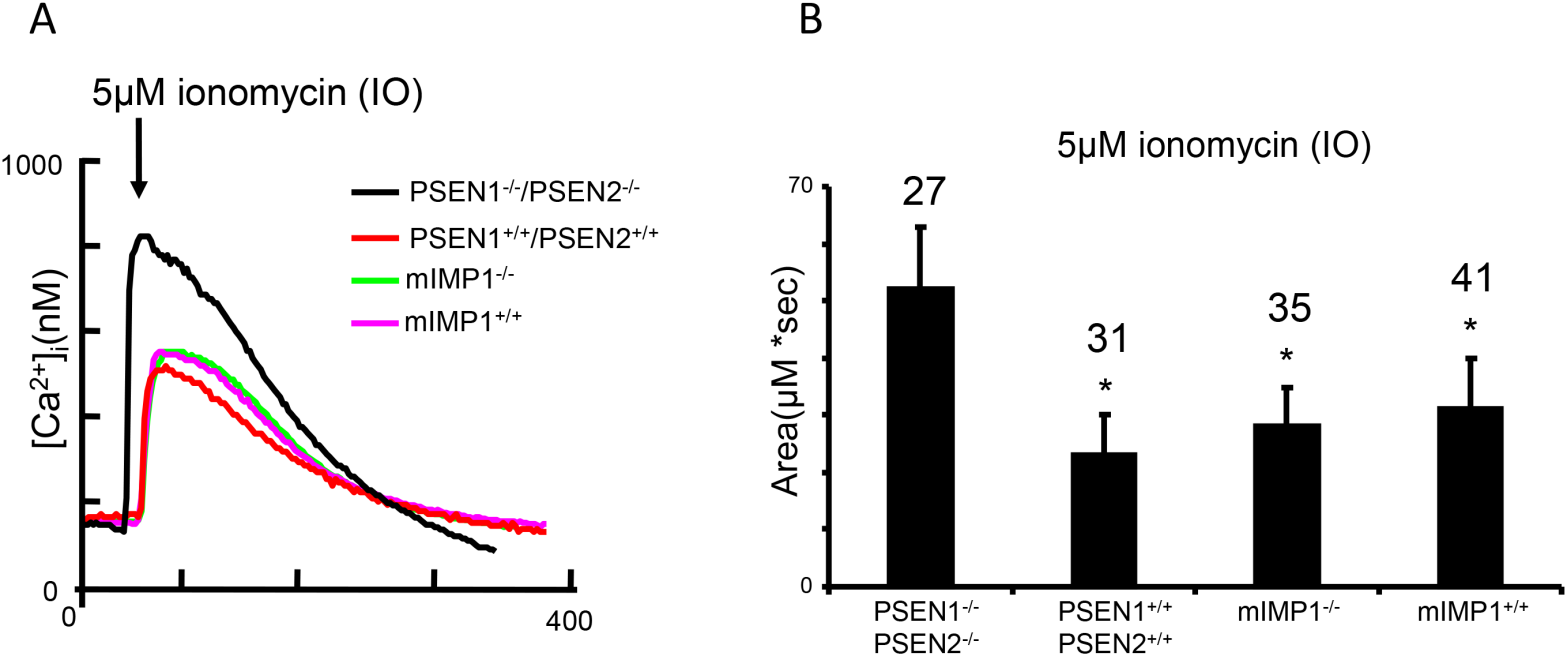
Low conductance ER Ca^2+^ leak assay demonstrates that IMP1, unlike PSEN1, is not a ion channel **(A)** Ionomycin (IO)-induced Ca^2+^ signal curves in *PSEN1*^*-/-*^*/PSEN2*^*-/-*^, *mIMP1*^*-/-*^ and matching wild type control MEF lines. **(B)** The histogram presents the mean size of IO-induced Ca^2+^ pool (±SD) in *PSEN1*^*-/-*^*/PSEN2*^*-/-*^, mIMP1^-/-^ and matching control MEF lines. ‘*’ p<0.05 by ANOVA – significant Ca^2+^ pool changes compared to NC. The number of cells analyzed is indicated (n).

PSEN1 and IMP1/SPP are structurally related multipass proteins which cleave transmembrane domains of Type I and Type II proteins with opposite orientations [3, 43] (Figure 1). Whether the IMP1/SPP can also function as Ca^2+^ channels has never been studied. We addressed this question using cultured *mIMP1/SPP*^*-/-*^ MEFs obtained from mIMP1/SPP knockout mice that were generated in our laboratory (unpublished). We tested the Ca^2+^ flow in mIMP1/SPP^-/-^ and wild type mIMP1/SPP^+/+^ MEF cells in comparison to *PSEN1*^*-/-*^*/PSEN2*^*-/-*^ and wild type PSEN1^+/+^ MEF cells. In contrast to PSEN1 knockout cells, the absence of IMP1/SPP protein does not affect the ER Ca^2+^leak function, indicating that IMP1/SPP is not a Ca^2+^ pore (Figure 10).

### *In vivo* rescue experiments in *C. elegans*

*C. elegans* is a popular and useful model organism for understanding the conserved mechanisms of AD-related presenilin/γ-secretase function and in identifying the components of the γ-secretase and signaling pathways regulated by presenilins [82]. Presenilin regulation of Ca^2+^ channel activities, similar to mammals, has been recently demonstrated in *C. elegans* [30].

**Figure 11:**
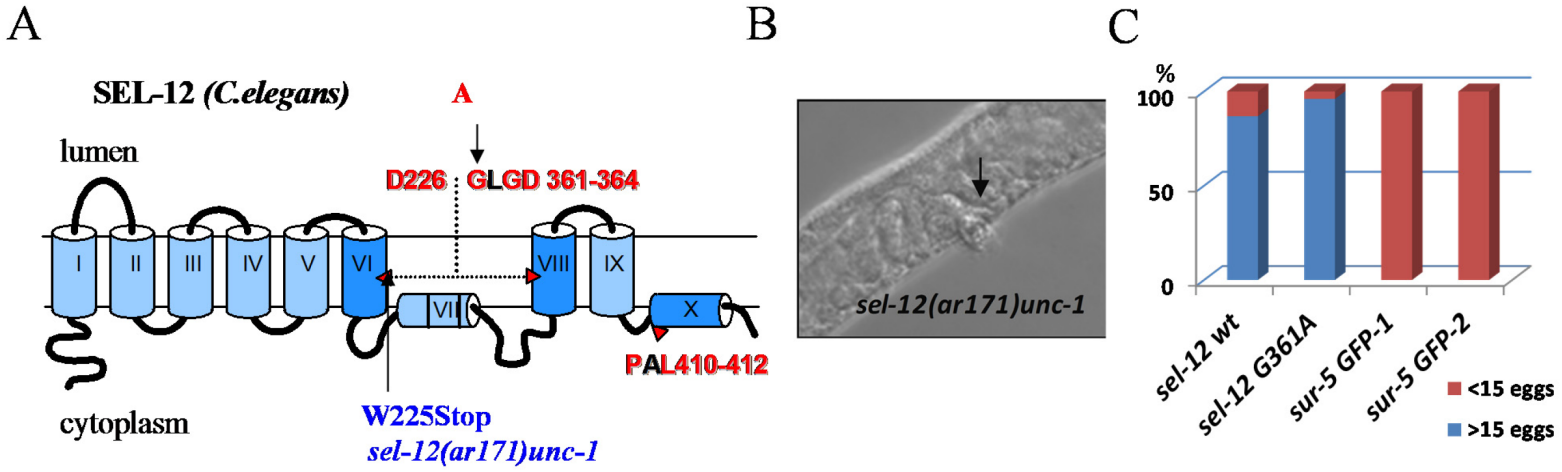
Rescue of *sel-12* loss-of-function mutant phenotype in *C. elegans* **(A)** Schematic representation of *C. elegans* SEL-12 protein (human PSEN1 homolog). The Sel-12 mutant isoform was generated with the G361A mutation (analogous to human G382A) introduced into the conservative amino acid motif of SEL-12. The *sel-12(ar171)unc-*1 strain contains a W225Stop mutation that results in truncated protein lacking domains that are critical for SEL-12 proteolytic function. **(B)** Egg-laying defective (Egl) phenotype in *sel-12(ar171)unc-1* strain. **(C)** The Egl phenotype is successfully rescued by injection of *sel-12* wt and *sel-12* G361A constructs but not by *sur-5 GFP* plasmid alone. Percentage of animals laying <15 or >15 eggs is shown. Number of analyzed animals for each strain: *sel-12 wt*, n=61; *sel-12* G361A, n=51; 2 control strains: *sur-5* GFP-1, n=50; *sur-5* GFP-2, n=50.

In the present study, we examined the *in vivo* effect of the mutation in PSEN1 (G382A) that does not suppress the ɛ-proteolytic activity, but inhibits the γ-secretase proteolytic and Ca^2+^ channel activities of PSEN1. We generated a *C. elegans* presenilin *sel-12* gene construct carrying a G361A mutation that corresponds to human PSEN1 position G382. We tested whether this mutant gene construct rescues the egg-lying defect (Egl) phenotype of *sel-12(ar171)unc-1 C. elegans* mutant, which has a premature stop codon and inactive Sel-12/presenilin protein [83]. We found that *sel-12* G361A, as well as *sel-12* wild type transgene expression can efficiently rescue the egg-lying defect (Figure 11). Li and Greenwald have described that reduction of the activity of another *C. elegans* presenilin homolog, *hop-1*, can cause lethality in *sel-12* mutant background strains [84]. Dead embryos/arrested larvae phenotypes are associated in general with a reduction of *lin-12*/*glp-1* Notch gene function, but does not exclude the influence of other signaling pathways [84]. In our experiments, s*el-12* G361A and sel*-12 wi*ld type strains, but not sur-5 *GFP cont*rols, can successfully survive for multiple generations being fed by hop-1 *RNAi* bacteria, proving the rescue effect of sel-12 G*361A i*n C. elegans *when both* presenilin homologues are suppressed (data not shown). All together, the data demonstrate that ɛ-proteolytic activity, but not the Ca^2+^ channel leak function, is essential for biological functions of presenilin, at least in early development and Notch-signaling.

**Figure 12:**
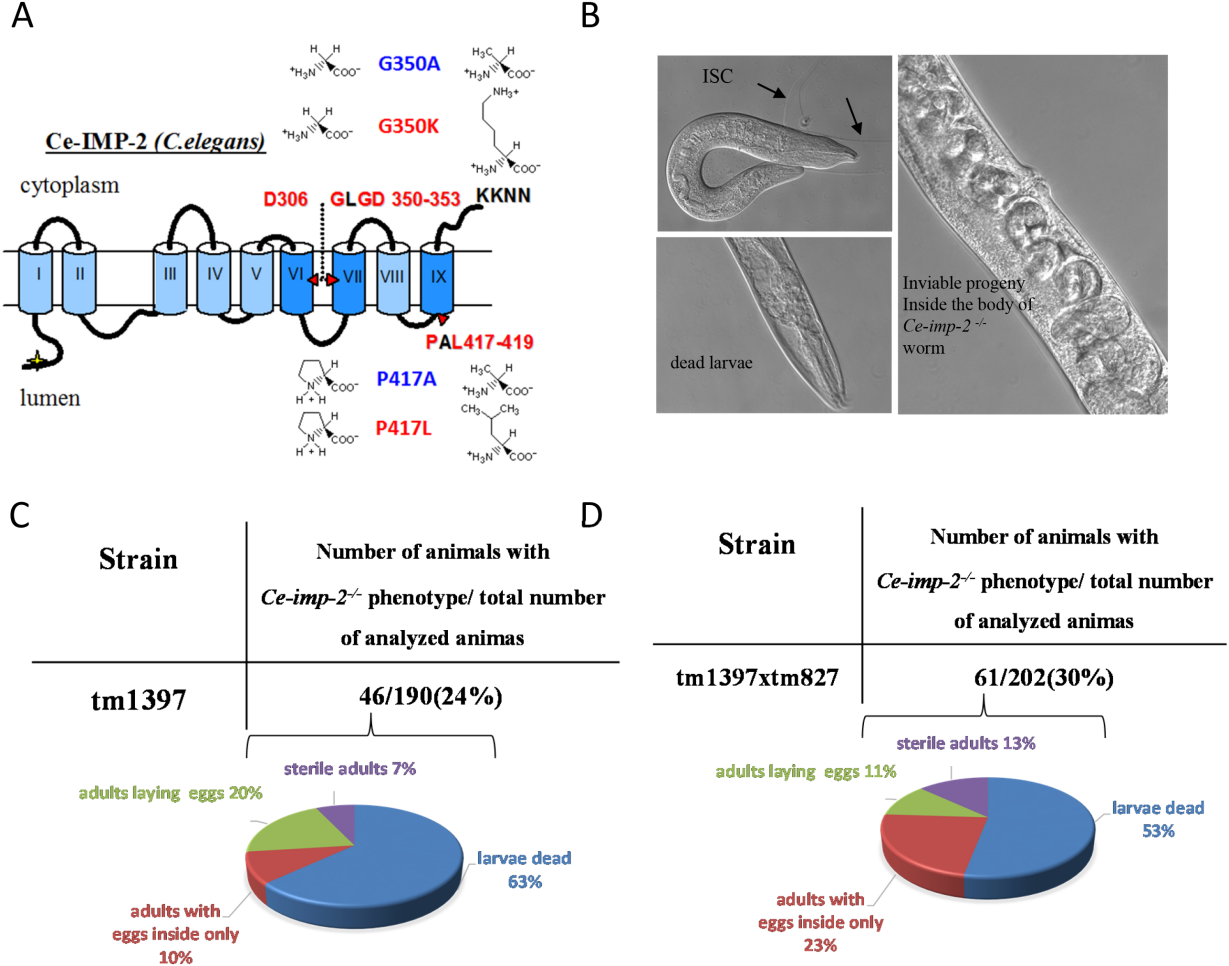
**(A)** *C.elegans* Ce-IMP-2 protein structure and mutations studied. Functionally important super-conservative sites (D306, GLGD 350-353, PAL 417-419) are indicated in red. **(B)** The loss-of-function of *Ce-imp-2* (homolog of human *SPP/hIMP1*) leads to two major defects: embryonic death and a larvae death/molting defect (black arrows) [48]. **(C, D)** Detailed analysis of phenotypes in progeny of *Ce-imp-2*^+/-^ (tm1397) and *Ce-imp-1*^-/-^/*Ce-imp-2*^+/-^ (tm827×tm1397) strains.

We described previously three genes in *C. elegans* (*Ce-imp-1, Ce-imp-2, Ce-imp-3*) that are homologous to five paralogous human genes (*IMP1/SPP* and *IMP-2, -3, -4, -5*) [42, 48]. Based on amino acid sequence alignments of these genes, we predicted that human hIMP1/SPP and *Ce-imp-2* are structural and functional orthologues (Figure 1B and 12A).

In cultured cells, both human IMP1/SPP and Ce-IMP-2 are capable of cleaving human PSEN1 holoprotein. Ce-IMP-2 had the high proteolytic efficiency, which is consistent with a functional relationship between hIMP1 and Ce-IMP-2 orthologous proteins (Figure 7A). To determine whether Ce-IMP-2 is capable of cleaving a C-terminal PSEN1 fragment, we expressed a C-terminal derivative (corresponding to the PSEN1 form processed by “presenilinase”) with hIMP1 and Ce-IMP-2 in double knockout mouse *PSEN1*^*-/-*^*/PSEN2*^*-/-*^ cells. The efficient cleavage of the C-terminus fragment was in the same domain and likely in an identical site that we described in the PSEN1 holoprotein (Figure 7B).

We reported previously that inhibition of the *Ce-imp-2* gene by microinjection of *Ce-imp-2* dsRNA or feeding worms with *E. coli*, producing *Ce-imp-2* dsRNA, resulted in slow growth, uncoordinated movement, reduced brood size, incomplete shedding of cuticle (ISC) as well as embryonic and larval death [48]. Here, we confirm these phenotypes in the *Ce-imp-2* knockout strain tm1397, bearing a 536-bp deletion of the promoter, exon 1 and part of exon 2, and the *Ce-imp-1 x Ce-imp-2* double knockout strain tm827×tm1397 (Figure 12B). We analyzed the F1 progeny of *Ce-imp-2*^+/-^ (tm1397) and *Ce-imp-1*^-/-^/*Ce-imp-2*^+/-^ (tm827×tm1397) worms and described the phenotypes of single or double knockouts (*Ce-imp-2*^-/-^ or *Ce-imp-1*^-/-^/*Ce-imp-2*^-/-^), confirmed by genotyping. The major observed phenotype was larvae death, about 30% of animals were able to generate eggs, but all the F2 progeny was inviable (Figure 12C, 12D).

**Figure 13:**
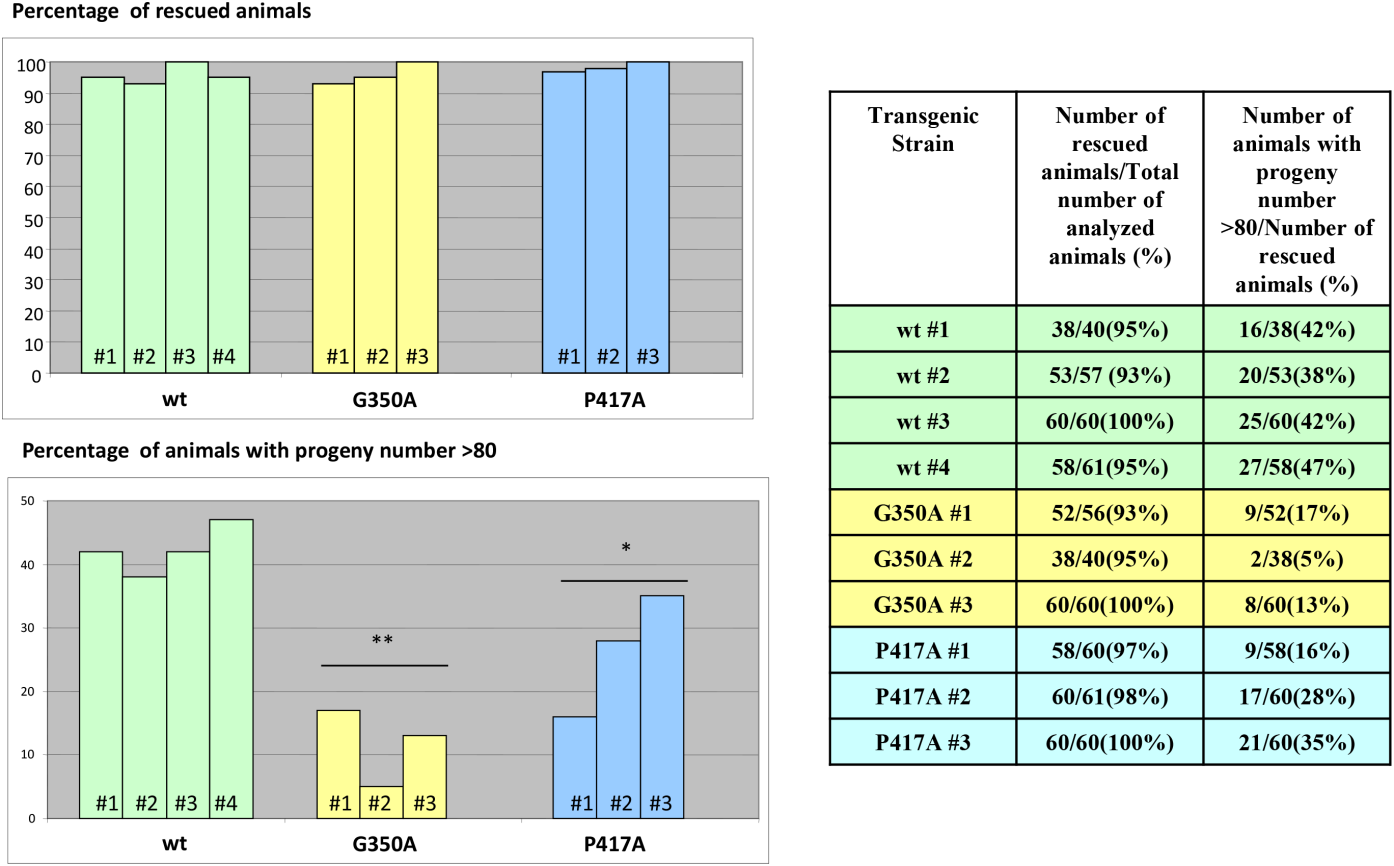
Rescue of *Ce-imp-2* knockout phenotype in *C. elegans* The constructs producing Ce-IMP-2 wild type and several mutant isoforms were generated. Several independent viable transgenic strains on *Ce-imp-2*^-/-^ (tm1397) background were obtained by picking 20-30 roller phenotype animals (pRF4 was used as a co-marker) from injected *Ce-imp-2*^+/-^ worm progeny. Rescue of *Ce-imp-2* knockout phenotypes was analyzed in 40-61 animals for each transgenic *Ce-imp-2*^-/-^ strain. Worms that can reach adult stage and have viable progeny were considered as rescued. The loss-of function *Ce-imp-2* phenotype was rescued by G350A, P417A Ce-imp-2 constructs.

**Figure 14:**
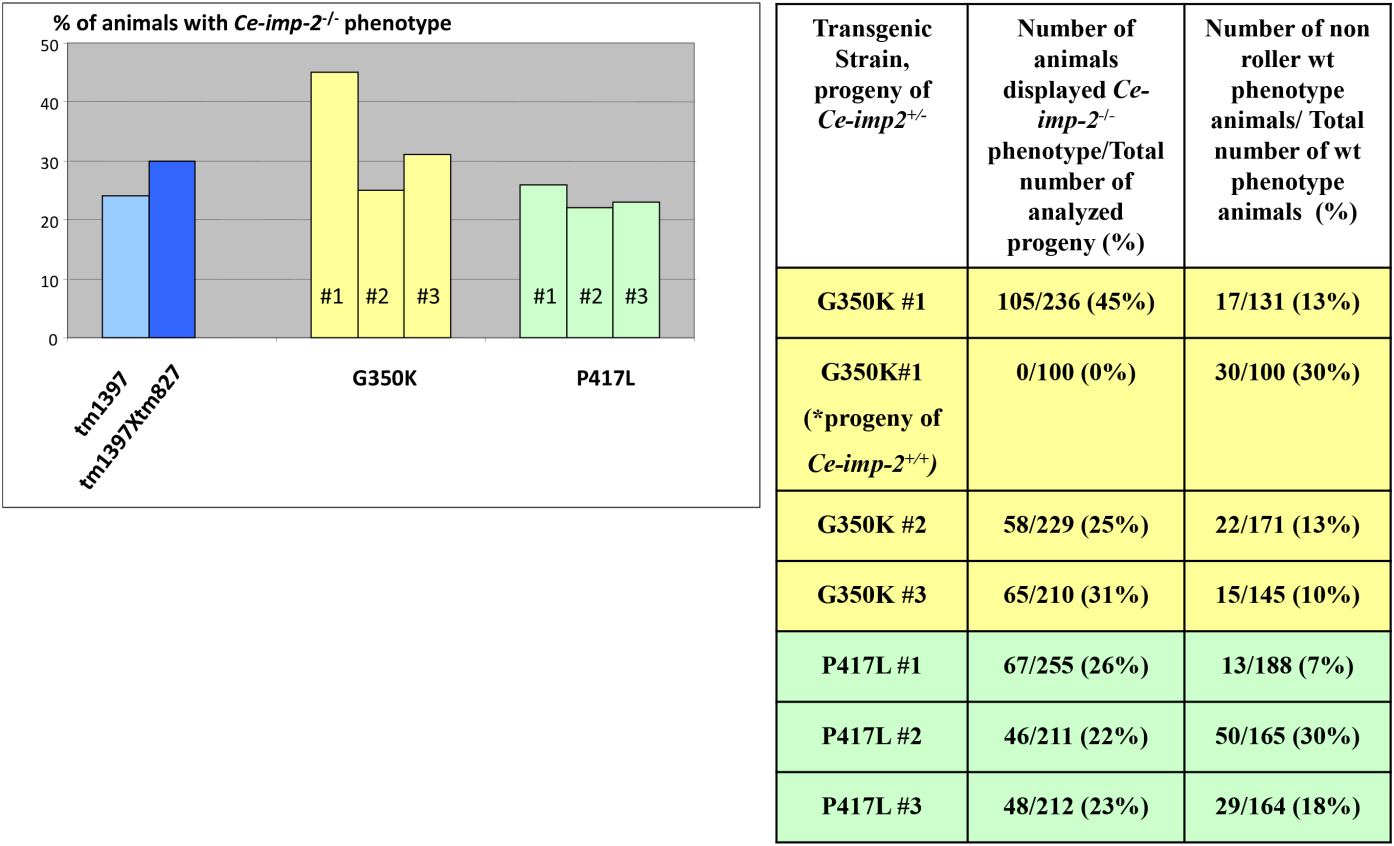
No rescue of *Ce-imp-2* knockout phenotypes was found by constructs producing Ce-IMP-2 G350K, P417L mutant proteins If none of the 20-30 roller phenotype (transgenic marker) worms, progeny of injected *Ce-imp-2*^+/-^ worms, displayed rescue phenotype on *Ce-imp-2*^-/-^ background, then ∼200 single worms, synchronized mixed viable progeny of several transgenic *Ce-imp-2*^+/-^ animals, were analyzed. In addition, for G350K strain #1, we checked phenotypes of *Ce-imp-2*^+/+^ animals progeny. The rate of the losing transgenic status was estimated as percentage of non-roller wild type phenotype animals/total number of wild type phenotype animals. P-value significance codes: ‘**’ <0.01, ‘*’ <0.05.

We then tested the effect of mutations in the *Ce-imp-2* gene in the rescue assays performed in the tm1397 *Ce-imp-2* knockout strain (Figures 12, 13; Supplementary Figure 5). *C. elegans* G350A and P417A mutations, corresponding to human G262A and P317A, successfully rescued the embryonic lethality on a *Ce-imp-2*^-/-^ background (Figure 13), although the progeny number was overall lower compared to the *Ce-imp-2* wild type construct (Figure 13). Mutations with distant physicochemical properties G350K and P417L showed no ability to rescue the mutant phenotypes compared to *Ce-imp-2* single and *Ce-imp-1 x Ce-imp-2* double knockout strains (Figure 14).

## DISCUSSION

Uncoupling the AD-related and physiologically important functions of presenilins represents a promising approach in the search for novel strategies for AD therapy. Genetically inherited forms of PSENs bearing the AD mutations most likely do not effect the early development of the organism, but rather, accumulate deleterious effects throughout the lifetime of the individual. Less dramatic early effects of the mutations on CNS function and development, however, cannot be excluded [85, 86]. Evolutionary analysis of two distantly related families of PSEN and IMP/SPP intramembrane di-aspartyl proteases shows a highly conserved amino acid signature around invariant aspartates in two transmembrane domains and the PAL motif at the C-terminal domain (Supplementary Figure 1). Here, we studied whether PSEN1 amino acid substitutions in the conserved regions of the protein have differential effects on PSEN1 proteolytic and non-proteolytic functions (Supplementary Figure 2). A similar mutational remodeling analysis was performed for the IMP1/SPP protein, which belongs to the family of evolutionarily ancient proteins distantly homologous to presenilins.

Previous *in vivo* and *in vitro* studies have shown that aspartates at positions 257 and 385 of PSEN1 play an essential role in γ-secretase proteolysis [4, 6, 83, 87–89]. These results were confirmed in our study.

The C-terminal proline 433 of PSEN1 has been described as being critical for γ-secretase function, participating in stable complex formation and catalytic pore structure [90-94, 28]. Our data demonstrate that substitutions of the conserved proline to structurally distant, but not to similar amino acids, suppress the proteolytic activity of PSEN1. However, PSEN1 with any mutation in this position can restore the normal Ca^2+^ leak function in PSEN1 knockout cells [28].

Mutation of the conserved leucine 435 of PSEN1 was found in familial cases of early onset AD with the unusual phenotype of cotton wool plaques [95, 96]. In our study, substitutions to structurally distant amino acids in this position have a prominent inhibitory effect on proteolytic function of PSEN1 with complete inhibition of Notch1 proteolysis and a strong inhibition of APP ɛ-proteolysis. Presenilinase function was also inhibited by L435D mutation, but to a lesser extent. Another unusual phenotype for AD, “Lewy bodies,” is associated with deletion of threonine 440 [97, 98]. We have shown that both PSEN1 T440D and T440V support cleavage of various substrates.

PSEN1 with a G382A mutation in the highly conserved motif of the second aspartate does not change physiologically essential proteolytic properties of the protein. The PSEN1 G382A mutation was functionally active in presenilinase, Notch and APP ɛ-proteolytic assays. Transgenic *C. elegans* experiments showed a successful rescue of an egg-laying defect linked to Notch proteolysis by the G361A Sel-12 construct. Distant mutations G382 K/L/Q/V completely abolished Notch1 and APP ɛ-site proteolysis and have a strong inhibitory effect on presenilinase cleavage. The G382 position is critical for supporting normal Ca^2+^ signaling, as none of the mutants at this position were able to restore normal Ca^2+^ leak function (Figure 9 and [28]). The most unexpected results were obtained in the Aβ peptide assay. While PSEN1 G382L lacks both ɛ- and γ-proteolytic activities and does not produce any Aβ peptides, the PSEN1 G382A mutant retains the ɛ-cleavage activity, but significantly reduces the total production of Aβ peptides. In the reported data the occurrence of Aβ40 and Aβ42/43 fragments was detected for the G382A PSEN1 mutant in HEK293 cells expressing APP with the Swedish mutation [99]. HEK293 cells have a substantial endogenous PSEN1 activity [48]. In our study, we used a *PSEN1, PSEN2* double knockout MEF model for expression of the mutant PSEN isoforms, thereby excluding any effect of endogenous PSEN activity. We also used a single-amino acid high resolution PAAG assay and noticed the minor production of a novel Aβ-peptide form that does not correspond to Aβ40 or Aβ42. We demonstrated that the PSEN1 G382A mutation retains Notch1-proteolytic activity, which has also been described in [99]. However, PSEN1 G382A was reported to block the processing of the cell membrane glycoprotein CD44 [99]. Nonetheless, our data revealed that Sel-12 G361A transgenic worms with no endogenous *sel-12* presenilin activity are viable for multiple generations, providing further evidence that proteolytic activity of PSEN in processing of Notch is an essential biological function of PSEN.

Here we showed that both *C. elegans* Ce-IMP-2 and human hIMP1/SPP proteins are capable of cleaving the same multipass transmembrane protein (PSEN1-holoprotein and -CTF) in co-transfection experiments using mammalian cells (Figure 7). Interestingly, Ce-IMP-2 induced cleavages with efficiency higher than those observed for hIMP1. Despite strong structural similarities, the Ce-IMP-2 and hIMP1 proteins have differences in some domains, particularly at the N-terminus. We also observed that human IMP1/SPP likely forms dimeric complexes, while the *Ce-imp2* protein homolog does not (data not shown). Altogether, this may explain the difference in efficiency of cleavage of the same substrate between *C. elegans* and human IMP/SPP homologs. The data also indicate that *Ce-imp-2* is a true orthologue of human *IMP1/SPP*.

We also searched available SNP databases to identify if any mutation in PSEN1 and hIMP1/SPP that we studied can be found in common human populations. We have not identified such SNPs in more than 2,500 human genomes in the 1000 Genomes Project catalog [100]. The Ensemble variants database contains reports for PSEN1 AD-mutations M146V, C410Y and also AD-linked mutations in protein positions 284 and 435, which differ from the mutations in this position described in our paper. Interestingly, the PSEN1 D257N somatic mutation, leading to PSEN1 haploinsufficiency, has been found in lung carcinoma" (Supplementary Table 1). Amino acid substitutions in positions 245 and 319 of hIMP1 were found in malignant melanoma and endometrium carcinoma correspondingly (Supplementary Table 2). Thus, it would be of interest to elucidate further the potential role of these proteases in cancer.

In summary, we confirmed that any mutations at functionally essential aspartates of members of two distantly related families of intramembrane proteases, PSEN1 and IMP1, completely abolish all their proteolytic activities. Mutations in other highly conserved sites reduce, but do not completely suppress, at least some of the proteolytic activities of PSEN1. Substitutions to structurally distant amino-acids in the most conserved sites most dramatically change efficacy of proteolytic cleavages. Amino acid substitutions in less conserved sites (e.g. P284 and T440 in PSEN1) can reduce substrates proteolysis, but to a much lesser extent. For PSEN1, among different proteolytic assays, “presenilinase” activity is the most resistant to all mutation modifications in highly conserved sites. The PSEN1 G382A mutant retains the functionally important Notch1 and APP ɛ-cleavage activities but has reduced APP γ-cleavage activity and does not support the Ca^2+^ leak function, but rescues the lethality of *C. elegans Ce-imp-2* knockout. We also provided evidence that IMP1/SPP is not a Ca^2+^ channel as described for the structurally related PSEN1.

The important finding is that a single amino acid alteration in transmembrane domain 7 of PSEN1 can reduce the AD-related intramembrane γ-secretase activity but retain the biologically important juxtamembrane proteolytic function of PSEN1. These data raise the idea that uncoupling AD- and biologically-essential functions of presenilins may represent a promising novel therapeutic approach.

## MATERIALS AND METHODS

### Plasmid constructs for expression in mammalian cell cultures

Human IMP1 (hIMP1) and PSEN1 (hPSEN1) wild type constructs were described previously [47]. A QuikChange^®^ Site-Directed Mutagenesis Kit (Agilent Technologies Inc., Santa Clara, CA, USA) was used to incorporate mutations in the hIMP1 and hPSEN1 genes (Supplementary Tables 1 and 2). PSEN1 D385A and APP Swedish mutant (APP695ΔNL) constructs were obtained from the Mayo Clinic (Mayo Clinic, Jacksonville, FL). mNotch1 (myc-tagged NotchΔE and NICD) constructs were kindly provided by Dr. R. Kopan (Washington University School of Medicine, St. Louis, MO, USA). pcDNA Flag-HCVcore-HA plasmid was a gift from Dr. Kohji Moriishi (Osaka University, Osaka, Japan). QuikChange^®^. pcDNA4/myc-His B or pcDNA3 plasmids from Invitrogen (Thermo Fisher Scientific, Waltham, MA, USA) were used as mock controls. cDNA clones for *Ce-imp-1*, *Ce-imp-2*, and *Ce-imp-3* genes were obtained from Y. Kohara (Genome Biology Lab, National Institute of Genetics, Mishima, Japan) and cloned into pcDNA4/myc-His B plasmid (Invitrogen, Thermo Fisher Scientific) for the experiments performed in mammalian cell cultures.

### Mammalian cell cultures

Knockout *PSEN1*^*-/-*^*/PSEN2*^*-/-*^ and control *PSEN1*^*+/+*^*/PSEN2*^*+/+*^ mouse embryonic fibroblasts (MEF) were a gift from Dr. De Strooper (Center for Human Genetics, Katholieke Universiteit Leuven, and Flanders Interuniversity Institute for Biotechnology, Leuven, Belgium).

HEK293 and MEF cells were cultured in DMEM (Gibco, Thermo Fisher Scientific) containing 10% FBS (Hyclone Laboratories, Logan, UT), 2 mM L-glutamine at 37°C and 1% penicillin/streptomycin, 5% CO_2_. Primary cultures of MEF were generated from 13.5 dpc hIMP1 knockout embryos and immortalized by continuous passaging (unpublished).

### Transfection of mammalian cell lines and immunoblotting

Transient transfections were performed using Lipofectamine Plus Reagent (Invitrogen, Thermo Fisher Scientific) according to the manufacturer’s instructions. After 24-48 hours of transfection, cells were briefly washed 2 times in cold PBS, lysed in modified RIPA-buffer (50 mM Tris-HCl, pH 7.4, 1% NP-40, 0.25% deoxcholate Na, 150 mM NaCl, 1 mM EDTA) supplemented with Complete Mini Protease Inhibitor Cocktail (Roche Diagnostics, Indianapolis, IN) for 15 min at 4°C and subsequently centrifuged at 14,000 rpm for 10 min at 4°C. A NOVEX mini-cell gel electrophoresis system (Invitrogen, Thermo Fisher Scientific) was used for protein separation and electrotransfer procedures. Cell lysates (10-20 μg of total protein) or 10 μl of conditioned culture medium were mixed with 5x Laemmli sample buffer with reducing agent (1x sample buffer composition: 60 mM Tris-Cl pH 6.8, 2% SDS, 10% glycerol, 5 % β-mercaptoethanol, 0.01% bromophenol blue) centrifuged at 12,000 rpm for 5 min with or without prior boiling for 5 min, and loaded onto SDS PAAG mini-gels (Invitrogen, Thermo Fisher Scientific). Prestained molecular weight markers (Invitrogen, Thermo Fisher Scientific) were loaded into a separate well. Electrophoresis was run either in 10% (PSEN1, HCV detection) or 8% (Notch detection) Tris-Glycine PAAG or in Novex^®^ 10-20% Tricine Gel (for APP-CT detection) in 1X SDS running buffer at 125 V. After electrotransfer, Immobilon-P PVDF membranes (EMD Millipore, Billerica, MA USA) were washed in TBS-T buffer (50 mM Tris-HCl, pH 7.4, 150 mM NaCl, 0.05% Tween 20) 1 time for 10 min and 3 times for 5 min, incubated in blocking buffer (5% milk in TBS-T) at room temperature for 1 hour and placed in 10 ml of hybridization buffer (1% milk in TBS-T), containing primary antibodies in 1:500 – 1:5000 dilution, at 4°C overnight. After incubation with appropriate secondary antibodies, signal visualization was performed using an ECL Western blotting detection reagent kit and exposure to an X-ray film or via image capturing using a VersaDoc 5000 imaging system (Bio-Rad Laboratories, Hercules, CA). Experiments were replicated multiple times ( ≥3).

Polyclonal rabbit antibodies against hPSEN1 N-terminal polypeptide and CTF were described previously [47]. Antibody against c-myc epitope for IMP1 and Notch detection was obtained from Invitrogen (Thermo Fisher Scientific). The released APP intracellular fragments were detected in supernatants using polyclonal APP CTF antibody (Sigma-Aldrich). For detection of Aβ peptides, mAb 1E8 (Bayer-Schering Pharma AG, Berlin, Germany or provided by T. Dyrks, Schering, Berline, Germany) was used while mAb 22C11 was used for detection of N-terminal APP fragments (Chemicon, EMD Millipore). For HCV detection, monoclonal mouse anti-FLAG (Sigma-Aldrich) was used.

### Cell-free intramembrane cleavage assay

For intramembrane cleavage assays, *PSEN1*^*-/-*^*/PSEN2*^*-/-*^ MEF cells were transfected with either APP695ΔNL or NotchΔE and one of the PSEN1 isoforms or mock plasmid. After 48 hours of transfection, cells were washed twice with ice-cold PBS, scraped off the dishes, resuspended in 0.5 ml of hypotonic homogenization buffer (10 mM HEPES, pH 7.2; protease inhibitors cocktail), incubated on ice for 1 hour and frozen in liquid nitrogen. Frozen cells were thawed on ice for 1 hour and homogenized by passing through a 22-gauge hypodermic needle 5 times. Homogenates were centrifuged at 1000 at 4°C for 10 min and post-nuclear supernatants were saved. To isolate crude membranes, post-nuclear supernatants were subjected to centrifugation at 20000 g, 4°C for 45 min and the pellets were resuspended in assay buffer (150 mM Sodium Citrate, pH 6.4, 5 mM EDTA, protease inhibitors cocktail). Intramembrane cleavage was induced by incubation of the samples at 37°C for 2 hours. Reactions were stopped by placing samples on ice, and membranes precipitated by centrifugation at 16000 g, 4°C for 30 min. The released cleavage fragments were detected in supernatants and analyzed by immunoblot.

### Immunoprecipitation of Aβ secreted in cell culture media

After 24 hours of transfection, plates were washed twice with 1×DPBS and incubated for 24 hours in serum-free O-MEM. Conditioned media were collected, centrifuged at 3000 g for 10 min and supernatants were subjected to immunoprecipitation as described in [101]. Briefly, 400 μl of medium were mixed with 100 μl of 5x RIPA buffer (2.5% IGEPAL CA-630, 1.25% sodium deoxycholate, 0.25% SDS, 750 mM NaCl, 250 mM HEPES, pH 7.4) supplemented with 1 tablet of Complete Mini Protease Inhibitor Cocktail per 2 ml of 5x RIPA. Magnetic beads (25 μl; DynaBeads, Thermo Fisher Scientific) coupled with monoclonal antibody 1E8 (1 μg of mAb 1E8 per 1.68 × 10^7^ beads) per reaction were added. Samples were incubated on a shaker at 4°C overnight. After washing in PBS, 0.1% BSA three times and one time in 10 mM Tris-HCl, pH 7.4, Aβ peptides were eluted from the magnetic beads in sample buffer containing 0.36 M bis-Tris, 0.16 M N, N'-bis-(2-hydroxyethyl)-glycine, 1% SDS, 15% sucrose, and 0.004% bromophenol blue, 2.5% DTT at 95°C for 5 min.

### SDS-PAGE of Aβ peptides

Aβ peptides were separated in 0.5 mm thick 13% T%/5% C/8M urea bicine/bis-Tris/Tris SDS-PAGE minigels, (T%-total percentage concentration of acrylamide monomer, C% - the percentage of crosslinker relative to the total acrylamide monomer) [101]. Electrophoresis was for 2 hours at a constant current of 12 mA per gel at ambient temperature. Aβ peptides were transferred onto Immobilon-P PVDF membranes (Millipore, Billerica, MA USA) using a TE-70 ECL Semi-Dry Transfer Unit (GE Healthcare Life Sciences, Piscataway Township, NJ, USA) for 30 min at 1 mA/cm^2^ at room temperature as described in [102]. After transfer was completed, membranes were briefly washed in deionized water, boiled in 1 × PBS for 3 min in a microwave, blocked in Roti-Block blocking buffer (Carl Roth GmbH, Karlsruhe, Germany) and incubated with primary mAb 1E8 diluted 1:300 overnight at 4°C. After a series of washes in PBS-T buffer (1×PBS, 0.075% Tween 20), membranes were incubated with secondary anti-mouse horse radish peroxidase-conjugated antibody (Pierce Biotechnology, Thermo Fisher Scientific) 1:5000 for 1 h at room temperature, washed 3 × 10 min in PBS-T and subsequently developed for 5 min with ECL Advance reagents (GE Healthcare Life Sciences). Signals were detected using a VersaDock 5000 MP Imaging System, and analyzed using Quantity One 4.6.3 software (Bio-Rad Laboratories, Hercules, CA, USA). Along with bicine/bis-Tris/Tris SDS-PAGE, 10 μl of each sample of conditioned medium were analyzed by Tris-glycine PAAG electrophoresis-immunoblot with monoclonal 22C11 antibody, that recognizes full length APP. To normalize signals of Aβ peptides, serial dilutions of synthetic Aβ peptides (Aβ 1-37, Aβ 1-38, Aβ 1-39, Aβ 1-40, Aβ 1-42, Aβ 2-42, Bachem, Bubendorf, Switzerland) were used [103].

### *C. elegans* experiments

Standard methods of *C. elegans* handling and culture were applied [104]. N2 Bristol strain was used as the wild type. *Sel-12(ar171)unc-1 (e538)* was provided by I. Greenwald (Columbia University, New York, USA). Tm827 (*Ce-imp-1* knockout), tm1397 (*Ce-imp-2* knockout) and tm1654 (*Ce-imp-3* knockout) were provided by the Mitani Laboratory, Tokyo Women's Medical University School of Medicine, Japan and outcrossed 3-5 times to the N2 strain. A *C. elegans* ∼6 kb *sel-12* genomic fragment containing ∼3 kb of the 5'- and ∼0.8 kb of the 3'- regulatory regions were cloned into the L4440 vector. *Ce-imp-2* constructs are described in Supplementary Figure 5. Mutations in the *sel-12* and *Ce-imp-2* genes were introduced using a QuikChange^®^ Site-Directed Mutagenesis Kit (Stratagene, Agilent Technologies). RNA interference (RNAi) by dsRNA feeding and germ-line injections were performed as previously described [48]. *Sur-5 GFP* or *pRF4* plasmids were used as germ-line co-injection markers. *Ce-imp* knockout and transgenic strains were genotyped accordingly (Supplementary Figure 5, Supplementary Table 3). The progeny of the heterozygous deletion mutant *Ce-imp-2* were analyzed by single worm PCR.

### ER Ca2^+^ channel leak study

Experimental methodology for measuring ER Ca^2+^ channel leak was previously described [27, 28].

### Statistical analysis

Plotted values represent mean ± standard error of the mean. Comparisons of more than two groups were carried out using one-way ANOVA and unpaired T-test against WT values as the control group (Figure 2-4, 6, 8). For the analysis of total Aβ in PSEN1 mutants (Figure 5) and *C. elegans* experiments (Figure 13-14) WT values were converted to Z-score using mutant data and two-tailed P-value was calculated. In all cases P < 0.05 was considered to be statistically significant after adjusting for multiple testing with Benjamini & Hochberg procedure.

## SUPPLEMENTARY MATERIALS FIGURES AND TABLES


