# Supplementary material for: Mutational re-modeling of di-aspartyl intramembrane proteases: uncoupling physiologically-relevant activities from those associated with Alzheimer’s disease
Source: Oncotarget. 2017 May 30;8(47):82006–26. doi: 10.18632/oncotarget.18299 (PMC5669866; doi:10.18632/oncotarget.18299)
Supplement: Supplementary file 1 [file oncotarget-08-82006-s001.pdf]

Mutational re-modeling of di-aspartyl intramembrane proteases: uncoupling physiologically-relevant activities from those associated with Alzheimer’s disease

SUPPLEMENTARY MATERIALS

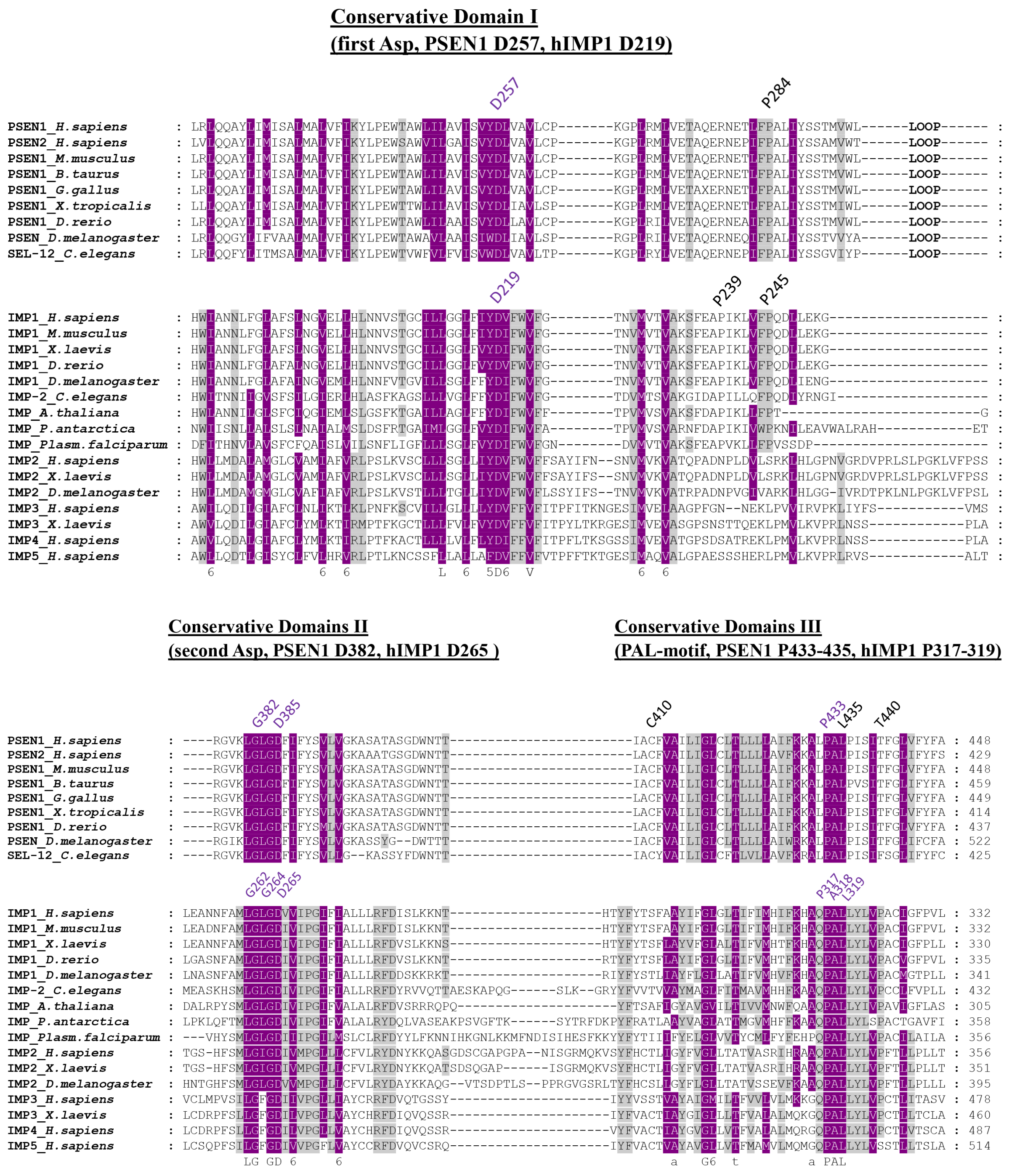

Supplementary Figure 1: Multiple sequence alignment of PSENs and distant presenilin homologous proteins (IMP 1-5, termed also SPP, SPPLs or PSH).

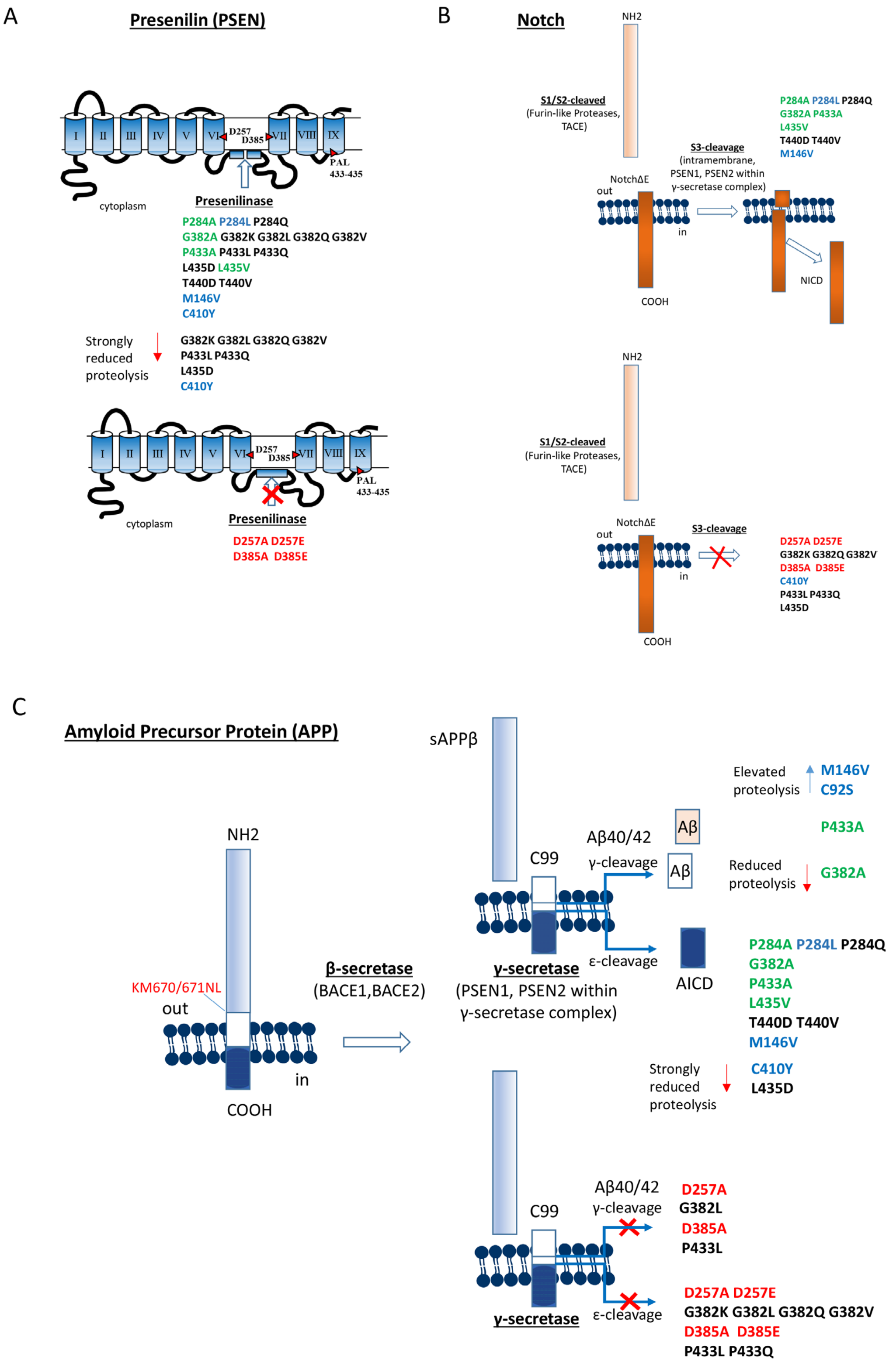

Supplementary Figure 2: (A, B). Schematic representation of mutations in Presenilin 1 affecting proteolytic activities of Presenilinase **(A)** and Notch S3 cleavage **(B)**. **(C)**. Schematic representation of mutations in Presenilin 1 affecting proteolytic activities of APP γ-secretase cleavage. APP695 isoform that bears the Swedish mutation for AD (KM670/671NL) was used in the experiments. In this and other Figures, mutations in super-conserved aspartates are indicated in red, mutations with similar amino acid changes, which do not alter the physicochemical properties of the site, are in green and AD mutations are in blue.

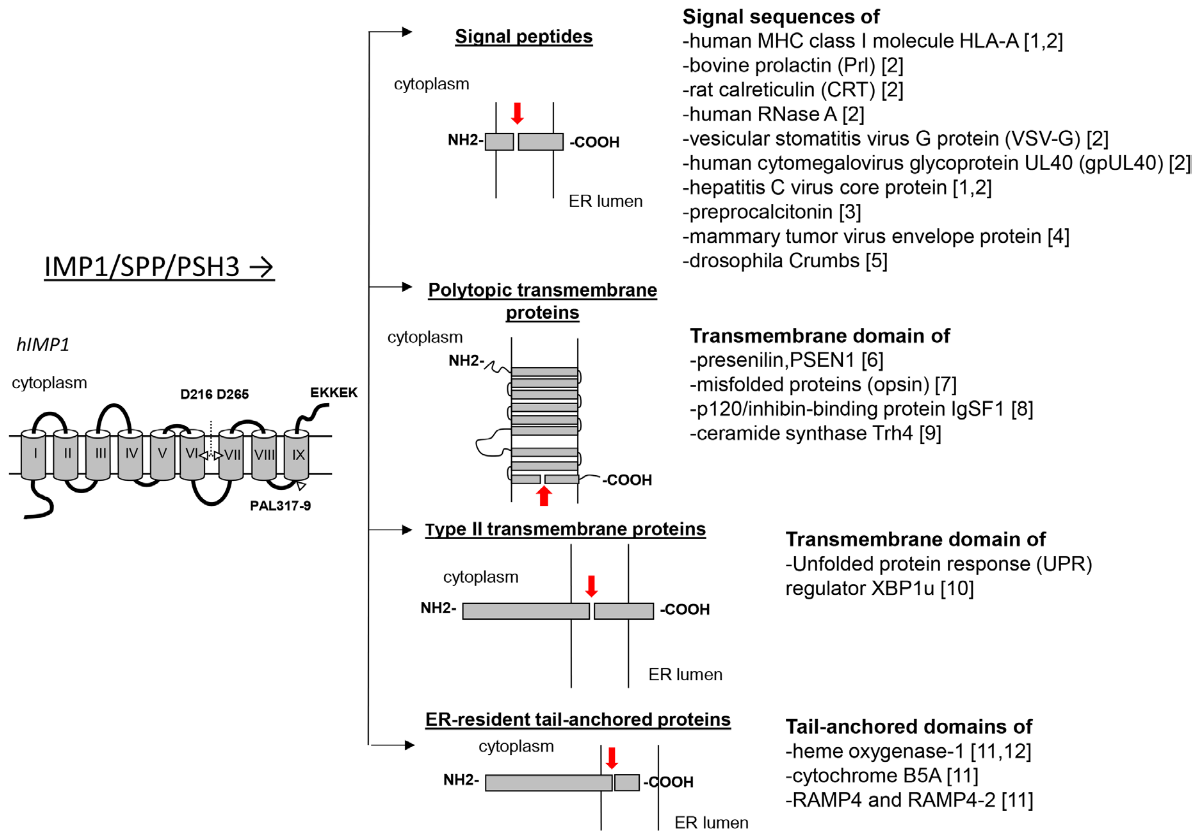

Supplementary Figure 3: IMP1/SPP substrates.

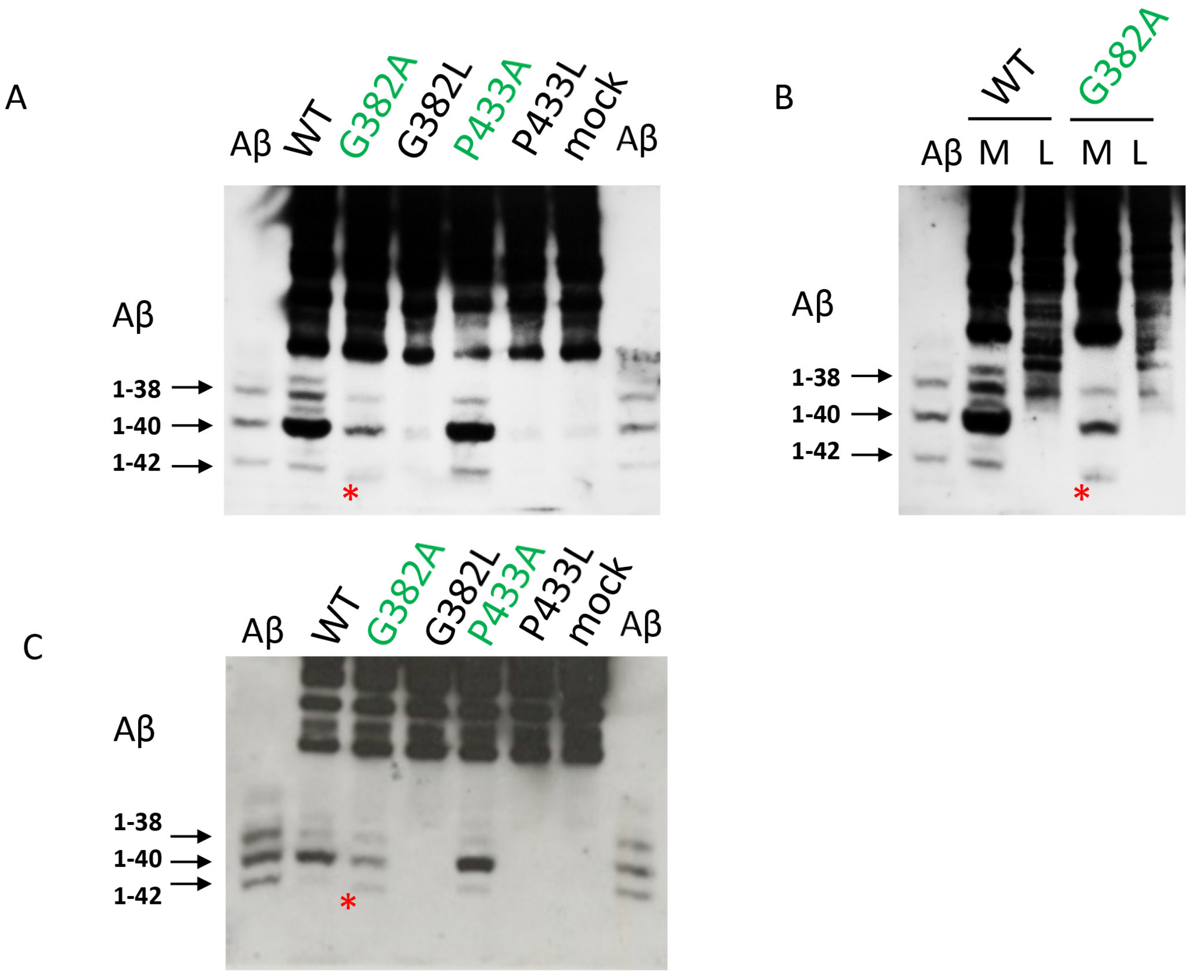

Supplementary Figure 4: Detection of secreted Aβ peptides in *PSEN1$^{-/-}$/PSEN2$^{-/-}$* MEF cells co-transfected with wild type or mutant PSEN1 and APP695ΔNL. Aβ peptides were detected in cell medium (**A, B** (M lanes) and **C**), but not in cell lysates (B - L lanes). **(A, B)** - immunoblots described in details in Figure 5 of the main text are shown with a higher exposure. Independent experiment **(C)** shows a specific Aβ peptide product (*), produced by the G382A mutant, which differs from Aβ42.

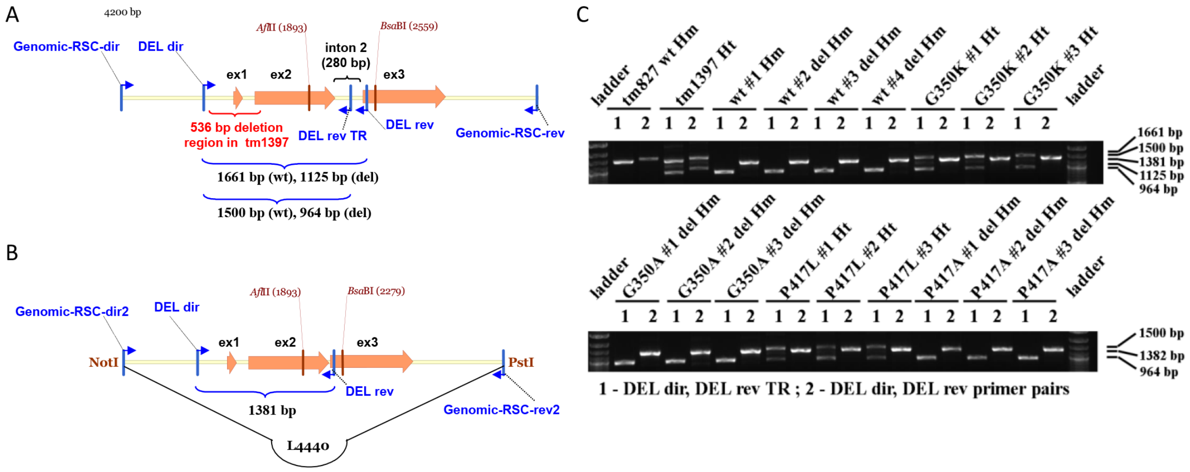

Supplementary Figure 5: Construct used for the rescue experiments in *C. elegans* and genotyping strategy. (A) Schematic representation of *Ce-imp-2* genomic region in wild type and tm1397 strains, bearing 536 bp deletion at the beginning of the gene. DEL dir, DEL rev and DEL rev TR primers were used for genotyping. **(B)** Plasmid construction used in *C. elegans* rescue experiments. Genomic fragment of *imp-2* gene was cloned into L4440 vector and AflII-BsaB1 genomic fragment was substituted with CDS fragment skipping intron 2. **(C)** Genotyping of worms. Two pairs of primers (1-Del dir/Del rev TR and 2- DEL dir/DEL rev) identify correspondingly: 1) 1500 bp and 1661 bp fragments in N2 wild type strain or *Ce-imp-1* knockout strain tm827; 2) 1500 bp, 964 bp and 1661 bp, 1125 bp fragments in *Ce-imp-2*$^{+/-}$ (Ht) worms of tm1397 strain ; 3) 964 bp and 1381 bp fragments in *Ce-imp-2*$^{-/-}$ (Hm) worm strains rescued with *Ce-imp-2* wild type (wt), G350A, P417A transgenes; 4) 1500 bp, 964 bp and 1381 bp fragments in *Ce-imp-2*$^{+/-}$ (Ht) worm strains with *Ce-imp-2* G350K and P417L transgenes. DEL dir/DEL rev 1125 bp genomic fragment amplification was inhibited by predominant amplification of transgenic 1381 bp fragment in all transgenic strains. Wt #1-4, G350K #1-3, G350A #1-3, P417L #1-3, P417A #1-3 are independent transgenic strains generated in the course of experiments.

SUPPLEMENTARY FIGURE REFERENCES

1. Weihofen A, Binns K, Lemberg MK, Ashman K, Martoglio B. Identification of signal peptide peptidase, a presenilin-type aspartic protease. Science. 2002; 296:2215–18.
2. Lemberg MK, Martoglio B. Requirements for signal peptide peptidase-catalyzed intramembrane proteolysis. Mol Cell. 2002; 10:735–44.
3. El Hage F, Stroobant V, Vergnon I, Baurain JF, Echchakir H, Lazar V, Chouaib S, Coulie PG, Mami-Chouaib F. Preprocalcitonin signal peptide generates a cytotoxic T lymphocyte-defined tumor epitope processed by a proteasome-independent pathway. Proc Natl Acad Sci USA. 2008; 105:10119–24.
4. Dultz E, Hildenbeutel M, Martoglio B, Hochman J, Dobberstein B, Kapp K. The signal peptide of the mouse mammary tumor virus Rem protein is released from the endoplasmic reticulum membrane and accumulates in nucleoli. J Biol Chem. 2008; 283:9966–76.
5. Kilic A, Klose S, Dobberstein B, Knust E, Kapp K. The Drosophila Crumbs signal peptide is unusually long and is a substrate for signal peptide peptidase. Eur J Cell Biol. 2010; 89:449–61.
6. Moliaka YK, Grigorenko A, Madera D, Rogaev EI. Impas 1 possesses endoproteolytic activity against multipass membrane protein substrate cleaving the presenilin 1 holoprotein. FEBS Lett. 2004; 557:185–92.
7. Crawshaw SG, Martoglio B, Meacock SL, High S. A misassembled transmembrane domain of a polytopic protein associates with signal peptide peptidase. Biochem J. 2004; 384:9–17.
8. Robakis T, Bak B, Lin SH, Bernard DJ, Scheiffele P. An internal signal sequence directs intramembrane proteolysis of a cellular immunoglobulin domain protein. J Biol Chem. 2008; 283:36369–76.
9. Oliveira CC, Querido B, Sluijter M, de Groot AF, van der Zee R, Rabelink MJ, Hoeben RC, Ossendorp F, van der Burg SH, van Hall T. New role of signal peptide peptidase to liberate C-terminal peptides for MHC class I presentation. J Immunol. 2013; 191:4020–28.
10. Chen CY, Malchus NS, Hehn B, Stelzer W, Avci D, Langosch D, Lemberg MK. Signal peptide peptidase functions in ERAD to cleave the unfolded protein response regulator XBP1u. EMBO J. 2014; 33:2492–506.
11. Boname JM, Bloor S, Wandel MP, Nathan JA, Antrobus R, Dingwell KS, Thurston TL, Smith DL, Smith JC, Randow F, Lehner PJ. Cleavage by signal peptide peptidase is required for the degradation of selected tail-anchored proteins. J Cell Biol. 2014; 205:847–62.
12. Hsu FF, Yeh CT, Sun YJ, Chiang MT, Lan WM, Li FA, Lee WH, Chau LY. Signal peptide peptidase-mediated nuclear localization of heme oxygenase-1 promotes cancer cell proliferation and invasion independent of its enzymatic activity. Oncogene. 2015; 34:2360–70.

SUPPLEMENTARY TABLES

Supplementary Table 1: List of mutated PSEN1 constructs. Amino acid changes with similar physicochemical properties are in green boxes [1, 2]

Construct PSEN1	WT amino acid	Mutated amino acid	Site position	Known AD mutations in this position, Alzforum.org	dbSNP 146 & COSMIC variants
M146V	hydrophobic	hydrophobic	AD mutation	**M146V,** M146I, M146L	M146L, M146V rs63750306 M146I rs63750391
D257A	**hydrophilic**	hydrophobic	First conserved aspartate	no	D257D rs200481721
D257E	**hydrophilic**	**hydrophilic**	First conserved aspartate		D257N COSM698729
P284A	special, imino acid	hydrophobic	Partially conserved proline (first **P**AL-motif in the hydrophobic part of the loop)	P284L, P284S	P284S rs63750324 P284L rs63750863
P284L	special, imino acid	hydrophobic	Partially conserved (first **P**AL-motif in the hydrophobic part of the loop)		
P284Q	special, imino acid	**hydrophilic**	Partially conserved (first **P**AL-motif in the hydrophobic part of the loop)		
G382A	special	hydrophobic	First conserved glycine within **G**LGD-motif of the second conserved aspartate	no	no
G382K	special	**hydrophilic**	First conserved glycine within **G**LGD-motif of the second conserved aspartate		
G382L	special	hydrophobic	First conserved glycine within **G**LGD-motif of the second conserved aspartate		
G382Q	special	**hydrophilic**	First conserved glycine within **G**LGD-motif of the second conserved aspartate		
G382V	special	hydrophobic	First conserved glycine within **G**LGD-motif of the second conserved aspartate		
D385A	**hydrophilic**	hydrophobic	Second conserved aspartate	no	no
D385E	**hydrophilic**	**hydrophilic**	Second conserved aspartate		
C410Y	special, SH	aromatic	AD mutation	**C410Y**	C410Y, C410F rs661
P433A	special, imino acid	hydrophobic	Conserved proline (second **P**AL-motif)	no	no
P433L	special, imino acid	hydrophobic	Conserved proline (second **P**AL-motif)		
P433Q	special, imino acid	**hydrophilic**	Conserved proline (second **P**AL-motif)		
L435D	hydrophobic	**hydrophilic**	Conserved lysine (second PA**L**-motif)	L435F	L435F rs63750001
L435V	hydrophobic	hydrophobic	Conserved lysine (second PA**L**-motif)		
T440D	hydrophobic	**hydrophilic**	Next to the second PA**L**-motif	T440del	no
T440V	hydrophobic	hydrophobic	Next to the second PA**L**-motif		

Supplementary Table 2: List of mutated hIMP1 constructs. Amino acid changes with similar physicochemical properties are in green boxes [1, 2]

Construct hIMP1	WT amino acid	Mutated amino acid	Site position	dbSNP 146 & COSMIC variants
D219A	**hydrophilic**	hydrophobic	First conserved aspartate	D219D rs149298851
D219E	**hydrophilic**	**hydrophilic**	First conserved aspartate	
P239L	special, imino acid	hydrophobic	Partially conserved proline (next to the first conserved aspartate)	no
P245L	special, imino acid	hydrophobic	Partially conserved proline (next to the first conserved aspartate)	P245S COSM3545056, COSM3545057
G262A	special	hydrophobic	First conserved glycine within **G**xGD-motif of the second conserved aspartate	
G262K	special	**hydrophilic**	First conserved glycine within **G**xGD-motif of the second conserved aspartate	
G262L	special	hydrophobic	First conserved glycine within **G**xGD-motif of the second conserved aspartate	no
G262Q	special	**hydrophilic**	First conserved glycine within **G**xGD-motif of the second conserved aspartate	
G262V	special	hydrophobic	First conserved glycine within **G**xGD-motif of the second conserved aspartate	
G264A	special	hydrophobic	Second conserved glycine within Gx**G**D-motif of the second conserved aspartate	no
D265A	**hydrophilic**	hydrophobic	Second conserved aspartate	D265D rs75266019, COSM5243623, COSM5243624,
P317A	special, imino acid	hydrophobic	Conserved proline (**P**AL-motif)	
P317L	special, imino acid	hydrophobic	Conserved proline (**P**AL-motif)	no
P317Q	special, imino acid	**hydrophilic**	Conserved proline (**P**AL-motif)	
A318C	hydrophobic	special , SH	Conserved alanine (P**A**L-motif)	no
L319D	hydrophobic	**hydrophilic**	Conserved lysine (PA**L**-motif)	L319H COSM1025580, COSM1592518
L319V	hydrophobic	hydrophobic	Conserved lysine (PA**L**-motif)	

Supplementary Table 3: List of primers used for *C. elegans imp-2* cloning and transgenic/knockout strains genotyping

C. elegans imp-2		
Genomic-RSC-dir	TCTTTTTGGAACACTTAATCCAG	
Genomic-RSC-rev	AGTCTTGGTTTTGGTAGCTTGC	
Genomic-RSC-dir2 (NotI)	TTTT-GCGGCCGC-AGCGAAAAACGAATTCGAAAG	
Genomic-RSC-rev2 (PstI)	TTTT-CTGCAG-AGTCTTGGTTTTGGTAGCTTG	
DEL dir	GACAGCTCAGAACACGTGGA	
DEL rev	TTCCGAAAACCCAGAAGATG	
DEL rev TR	CAGCTTTAGGCCAAAATTGA	
C. elegans imp-1		
tm827delF	TTGAAATTCGGTTCCATCGT	WT - 535 bp
tm827delR	CTCCACAAGTCGGAAACCAT	tm827 - 253 bp
C. elegans imp-3		
tm1654delF	GGTGGAATAATACCTGGTGGAA	WT - 866 bp
tm1654delR	AAAGAAGCGCCAATATCGAA	tm1654 - 146 bp

SUPPLEMENTARY TABLE REFERENCES

1. Frömmel C. The apolar surface area of amino acids and its empirical correlation with hydrophobic free energy. J Theor Biol. 1984; 111:247–60.

2. Livingstone CD, Barton GJ. Protein sequence alignments: a strategy for the hierarchical analysis of residue conservation. Comput Appl Biosci. 1993; 9:745–56.
